# Predicting Blood–Brain Barrier Permeability of Marine-Derived Kinase Inhibitors Using Ensemble Classifiers Reveals Potential Hits for Neurodegenerative Disorders

**DOI:** 10.3390/md17020081

**Published:** 2019-01-29

**Authors:** Fabien Plisson, Andrew M. Piggott

**Affiliations:** 1 CONACYT, Unidad de Genómica Avanzada, Laboratorio Nacional de Genómica para la Biodiversidad (Langebio), Centro de Investigación y de Estudios Avanzados del IPN, Irapuato, Guanajuato 36824, Mexico; 2 Institute for Molecular Bioscience, The University of Queensland, St. Lucia, QLD 4072, Australia; andrew.piggott@mq.edu.au; 3 Department of Molecular Sciences, Macquarie University, Sydney, NSW 2109, Australia

**Keywords:** marine natural products, kinase inhibitors, blood–brain barrier permeability, neurological disorders, machine learning, QSPR, RDKit

## Abstract

The recent success of small-molecule kinase inhibitors as anticancer drugs has generated significant interest in their application to other clinical areas, such as disorders of the central nervous system (CNS). However, most kinase inhibitor drug candidates investigated to date have been ineffective at treating CNS disorders, mainly due to poor blood–brain barrier (BBB) permeability. It is, therefore, imperative to evaluate new chemical entities for both kinase inhibition and BBB permeability. Over the last 35 years, marine biodiscovery has yielded 471 natural products reported as kinase inhibitors, yet very few have been evaluated for BBB permeability. In this study, we revisited these marine natural products and predicted their ability to cross the BBB by applying freely available open-source chemoinformatics and machine learning algorithms to a training set of 332 previously reported CNS-penetrant small molecules. We evaluated several regression and classification models, and found that our optimised classifiers (random forest, gradient boosting, and logistic regression) outperformed other models, with overall cross-validated model accuracies of 80%–82% and 78%–80% on external testing. All 3 binary classifiers predicted 13 marine-derived kinase inhibitors with appropriate physicochemical characteristics for BBB permeability.

## 1. Introduction

The discovery of bryostatin 1, a macrolide isolated from the bryozoan *Bugula neritina* [1], marks one of early inputs of marine bioprospecting to kinase drug discovery. The Yuspa and Pettit groups reported bryostatin 1 as the first marine-derived protein kinase C (PKC) modulator inhibiting the allosteric binding site for endogenous messengers (e.g., diacylglycerol) and oncogenic phorbol esters (e.g., 12-*O*-tetredecanoylphorbol-13-acetate) known to induce skin carcinogenesis [2]. In addition to being extensively tested, clinically, for various tumours [3,4], bryostatin 1 also appears promising in enhancing memory in animal models, and is now in Phase IIa for the treatment of Alzheimer’s disease at the Blanchette Rockefeller Neurosciences Institute [5]. Following in the steps of bryostatin 1, several drug discovery campaigns have been established over the years to reveal marine natural products potentially active against targeted kinases. Between 2008 and 2012, we collaborated with Spanish biotechnology company Noscira S.A. to explore the chemistry of Australia’s rich marine biodiversity. This partnership led to the discovery of a range of unique kinase-inhibiting secondary metabolites, including the lamellarins [6], ningalins [7], pyrrolo-aminoimidazoles/bromopyrroles [8,9] and trachycladindoles [10], among others.

Several recent reviews have showcased the important contribution of marine natural products to kinase inhibitor discovery. Skropeta et al. reviewed kinase inhibitors isolated from marine sponges since 1980 in a target-centric manner [11]. Liu and co-authors reviewed naturally occurring kinase inhibitors by chemical class and from wider occurrences (i.e., plants, soil bacteria, fungi and marine sponges) [12]. Others have focused on specific groups of marine-derived kinase inhibitors (MDKIs) such as lamellarins [13], kahalalides [14] and meridianins [15]. Most notably, Bharate and co-workers published a comprehensive review of 354 MDKIs and synthetic analogues with an emphasis on structure–activity relationships studies, lead optimisation and clinical evaluations [16]. These literature accounts clearly highlight the biological relevance and chemical diversity of MDKIs as a privileged source of unique scaffolds for new kinase drugs (KDs). As a cornerstone of this study, we conducted an independent literature review to identify 471 MDKIs within 35 years of marine bioprospecting (1982–2017), which includes most of the aforementioned natural products and their synthetic analogues.

Blood–brain partitioning and brain distribution are critical properties for drugs targeting the central nervous system. The blood–brain barrier (BBB) is one of three barriers from the central nervous system that limit or regulate molecular exchange at the interface between the blood and the neural tissue or its fluid spaces. The two other barriers are the choroid plexus epithelium, between blood and ventricular cerebrospinal fluid (CSF); and the arachnoid epithelium, between blood and subarachnoid CSF. The BBB is made of endothelial cells joined together by tight junctions that act as a physical barrier preventing hydrophilic molecules larger than 400 Da from moving through the paracellular route. Small gaseous molecules can freely diffuse through lipid membranes by passive diffusion. Several energy-dependent active transporters are localised on luminal and abluminal membranes, providing alternative routes of administration and elimination to molecules, including P-glycoprotein and the multidrug resistance-associated protein (MRP) family [17]. Abbott and co-authors reviewed that numerous neurological pathologies, like Alzheimer’s disease, induce the dysfunction of the BBB in early stages, such as the loss of tightness in brain endothelial junctions [18]. Current strategies in drug delivery target both passive and active routes. Various methods have been established for measuring brain uptake, with the early labour-intensive in vivo measurements on murine models and low-throughput in vitro techniques stimulating the development of more efficient in silico (computational) models. The advantages and limitations of in vitro, in vivo and in silico methods have previously been reviewed at length [17,19,20].

Computational methods are particularly useful to help the selection of drug candidates exposed to the CNS. These methods are more cost-effective and less time-consuming than acquiring data through in vitro and in vivo experiments. Modelling BBB permeability falls under the area of quantitative structure–property relationship (QSPR) studies. These predictive models are developed with the application of machine learning methods, such as simple linear regression or more complex artificial neural networks, to a collection of chemical compounds with known pharmacokinetic properties and physicochemical characteristics. QSPR models are commonly applied to predict absorption, distribution, metabolism, and excretion properties for drug candidates. The ability of a molecule to permeate the BBB is measured by the blood–brain partitioning coefficient (logBB), which is defined as the logarithm of the ratio of the concentration of drug in the brain and in the blood (logBB = log_10_[brain]/[blood]). The measure of unidirectional clearance from blood to brain across the BBB, known as BBB permeability–surface area product (BBB PS), has also been proposed as a more accurate alternative. However, the lack of publicly available data for BBB PS has favoured the use of logBB as the index of BBB permeability. Numerous QSPR models with logBB or BBB PS have been developed since early 1990s [19], and are continuously proposed [21] both as regression and classification models. Initial models used physicochemical properties such as log P_oct_, molecular polarisability, pKa of labile hydrogen atoms and McGowan molecular weight, although the difficulty in accurately calculating these properties from chemical structures has limited the applicability of such models. More recently, most models agree that the most significant physicochemical properties to affect drug accessibility to the brain are the topological polar surface area (TPSA), hydrogen bond donors (HBD) and acceptors (HBA), rotatable bonds, lipophilicity (cLogP), and molecular weight (MW). In a 2009 study, Chico and co-authors [22] have reviewed the status of kinase inhibitor drugs for cancer treatments that failed to translate into neurodegenerative disorders, due to their low/poor BBB permeability. The authors found that kinase inhibitor drugs had different molecular property trends to known CNS-penetrant small molecules (CPSMs). In 2012, Ghose and co-workers developed a recursive partition model with some property-based guidelines between CNS and non-CNS drugs [23]. However, some authors claimed that recent efforts in CNS drug discovery across the pharmaceutical industry would enrich the number of drug candidates to be evaluated in the clinic, like proteases and kinases inhibitors, which could change the profile of CNS drug properties [24]. Chemoinformatic and machine learning algorithms also provide powerful tools to navigate the challenges associated with the development of MDKIs (and natural products in general) as drugs, which is often hindered by limited accessibility to natural resources, complex synthetic routes and elusive protein–ligand interactions. Recent reviews by Rodrigues [25] and the Medina-Franco group [26] highlight the advances in computer-aided drug design and its applicability to reveal the full potential of natural products.

The purpose of this study was to evaluate previously reported MDKIs for their ability to pass the BBB. In light of the practicality, cost-effectiveness and time reduction of processing QSPR models, we chose to predict the BBB permeability of our 471 MDKIs alongside 448 CPSMs and 49 KDs. To encourage dissemination and further development, we generated our data, exploratory analyses and predictive models with the freely available open-source chemoinformatics platform RDKit and Python 3.6. We used 332 CPSMs with available in vivo logBB data to build these models (Appendix A). We explored several regression and classification models prior to focus on specific binary classifiers for further optimisation. We assessed the predictive ability and the applicability domain of our models using difference metrics, cross-validation as well as external validation. Finally, we measured the probability of CPSMs (without logBB values), KDs and MDKIs for BBB permeability with our best models. We believe this is the first time such models have been applied to a large dataset of marine natural products.

## 2. Results and Discussion

### 2.1. Exploratory Data Analysis: Similarities, Differences and Multicollinearity between Compounds

**Figure 1:**
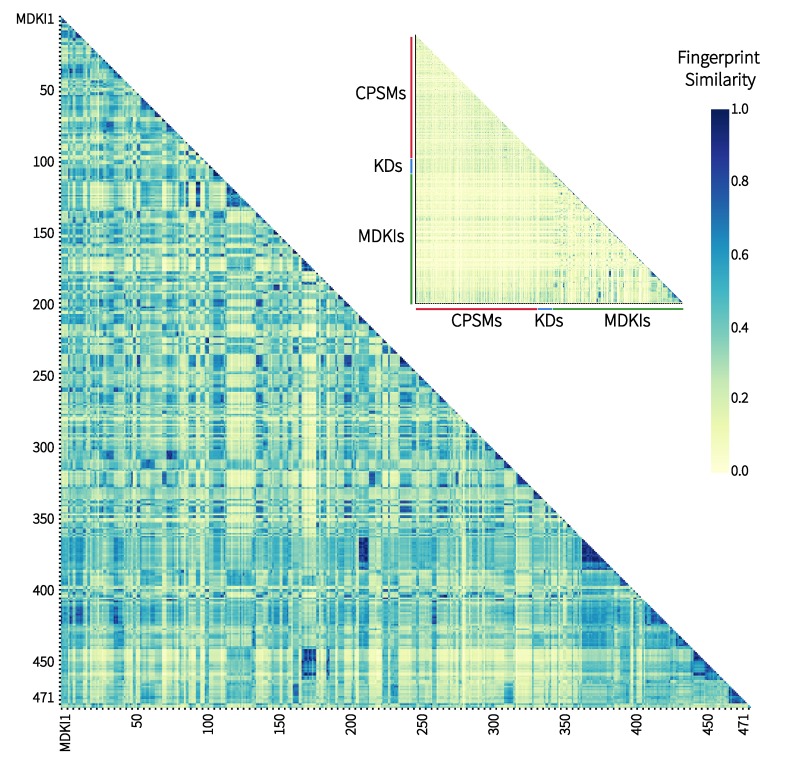
Fingerprint similarity matrix using public MACCS structural keys between the 968 structures grouped as central nervous system (CNS)-penetrant small molecules (CPSMs), kinase drugs (KDs) and marine-derived kinase inhibitors (MDKIs). The maximum similarity is observed at a value of 1.0 (dark blue).

Initially, we studied the molecular similarity between all 968 structures of the 3 datasets—448 CNS-penetrant small molecules (CPSMs), 49 kinase drugs (KDs) and 471 marine-derived kinase inhibitors (MDKIs), by either using their structural MACCS keys or their 200 physicochemical properties. With the former, we measured pairwise similarities between MACCS keys, molecular fingerprints of 166 characters in length, that were computed using open-source chemoinformatic software RDKit [27] into a symmetric matrix prior to colour-code these values in a two-dimensional heatmap, as illustrated in Figure 1. We have reproduced only half of the symmetric heatmap for ease of interpretation. A similarity value, known as ‘fingerprint similarity’, spans between 0 and 1, and is represented by a colour within a gradient from yellow to dark blue. The higher the value (i.e., the darker the colour), the more similar two structures are to each other. On the top right corner of Figure 1, we showed the entire dataset with CPSMs (red bar), KDs (blue) and MDKIs (green). Overall, we observed that the heatmap was mainly yellow, which indicates most compounds are structurally distant, with fingerprint similarities less than 0.5. It also suggested that our entire dataset was a collection of structurally diverse compounds. A magnified version of the small heatmap is available as Supplementary Figure S1a. We noted that small patches of dark blue were visible in the bottom right corner of that heatmap corresponding to the MDKI region. The region was magnified as the main heatmap in Figure 1. These patches corresponded to compounds of high to maximum similarity (above 0.8), which suggested that several MDKIs shared similar structures and common pharmacophores. This observation was not surprising as our literature search for kinase inhibitors of marine origin yielded several marine natural products belonging to the same structural classes (e.g., alkaloid) or groups such as the aforementioned meridianins [15]. These compounds were collected, sequentially, to form the MDKI dataset and, hence, they clustered into blue patches of different sizes. One of the largest patches observed along the diagonal at MDKI350–400 depicted the structural similarity of kinase inhibitors lamellarins (MDKI356–371) and ningalins (MDKI372–377) that we had previously reported [6,7]. That cluster demonstrated high fingerprint similarity to another group of MDKIs close to the MDKI200 mark; those are another group of lamellarins published by Meijer et al. in 2008 [28]. Finally, two other clusters of high similarity were centred around the MDKI150 and MDKI450 marks, corresponding to meridianins, isomeridianins and variolins (MDKI165–174), a rich group of ascidian-derived kinase inhibitors [29,30] that have led to the production of synthetic analogues (MDKI431–438) [31,32] and chimeric structures (meriolins; MDKI439–452) [33]. A magnified version of the heatmap is available as Supplementary Figure S1b.

**Figure 2:**
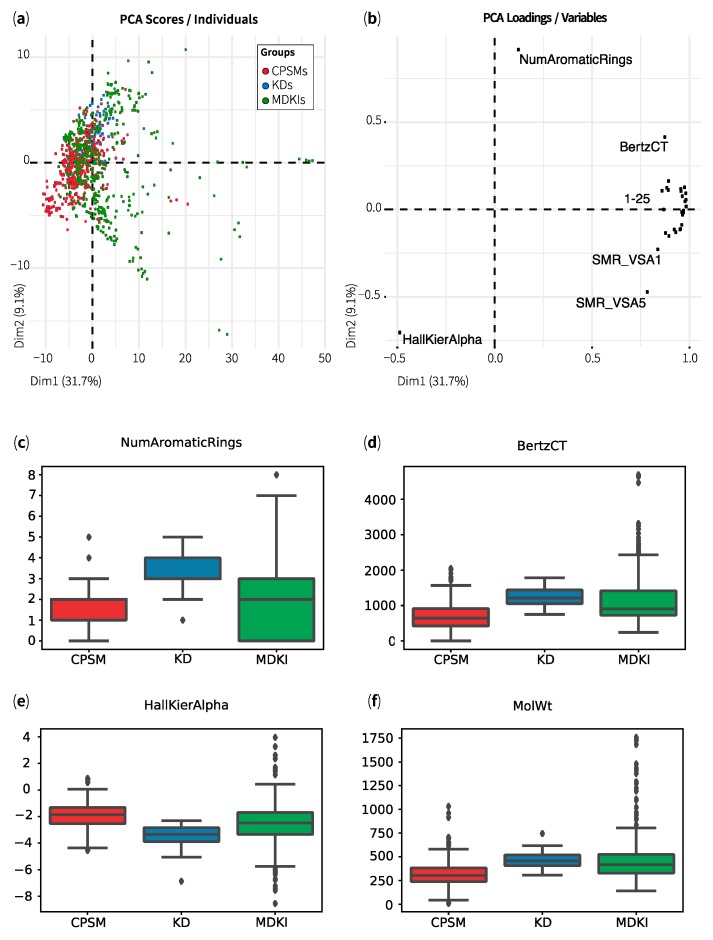
Distribution of CNS-penetrant small molecules (CPSMs, red), kinase drugs (KDs, blue) and marine-derived kinase inhibitors (MDKIs, green) along the first two principal components (PCA) with (**a**) scores displaying the 968 structures and (**b**) loadings representing the top30 contributing variables (1–25: Chi4v, Chi4n, Chi3v, Chi3n, Chi2v, Chi2n, Kappa1, Chi1n, Chi1v, Chi0v, Chi0n, NumValenceElectrons, Chi), LabuteASA, MolWt, ExactMW, HeavyAtomCount, HeavyAtomMolWt, MolMR, Chi1, VSA_EState9, NOCount, NumHAcceptors, NumHeteratoms, SlogP_VSA2). Boxplots showing mean values of four key variables: (**c**) number of aromatic rings, (**d**) an index of molecular complexity (BertzCT), (**e**) an index of shape (HallKierAlpha) and (**f**) molecular weight (MolWt). For the definition of all 200 variables, see Supplementary Materials.

Our second analysis of all 968 structures used a string of 200 physicochemical properties (herein referred to as “variables”) for each compound, which were calculated from three-dimensional lowest-energy conformations using RDKit [27]. The results were saved into a data frame of 968 observations by 200 variables. A complete list of the physicochemical descriptors is available as Supplementary Tables S1 and S2. In this analysis, we aimed to identify the structures that shared similar physicochemical properties and to visualise the 3 datasets—CPSMs, KDs and MDKIs—in property (chemical) space. The property space consisted of 200 dimensions, where each compound had 200 coordinates. After normalising the dataset, we applied principal component analysis (PCA) to convert all physicochemical properties into a set of linearly uncorrelated variables, known as principal components. The PCA scores (Figure 2a) described the new coordinates of all 968 structures, while the PCA loadings (Figure 2b) represented the physicochemical properties and their contributions to each transformation. In these figures, we presented only the first two principal components, which explained 31.7% and 9.1% of the total variance. The percentages of explained variance for the first 10 principal components can be seen in Supplementary Figure S2a. In Figure 2a, we noticed that most structures were located on the left hand of the plot; CPSMs (in red) spread over the extreme left, while KDs (barely visible, in blue) were clustered further to the right, on top of the red “cloud”. MDKIs (in green) occupied a wider distribution than CPSMs and KDs. Most KDs and many MDKIs clustered on the top region of the PCA because they contained more aromatic rings than most CPSMs (Figure 2b,c). In their comparative analysis, Feher et al. [34], followed by Grabowski and Schneider [35], observed that natural products, on average, contained fewer aromatic rings but more stereogenic centres than drugs. These observations remained valid when our KDs were compared to MDKIs (Figure 2c). In our comprehensive literature review, numerous MDKIs were identified as having multiple aromatic rings, in particular, fused-rings systems. Interestingly, CPSMs and most MDKIs exhibited higher Hall–Kier α indices than KDs (Figure 2e), leading to their clustering towards the bottom left corner of the PCA (Figure 2a). Hall–Kier α indices are indicative of high molecular connectivity between substructures such as aromatic rings, thus, MDKIs’ high values could be due to their fused-rings systems. Of note, Feher et al. [34] concluded that natural products were more rigid, partially due to these larger fused-rings systems. Figure 2b showed that several physicochemical properties strongly drove the distribution of compounds towards the right hand of the plot, in particular, for MDKIs outliers (beyond the main cluster). The strong contributors included various indices of shape and molecular connectivity (i.e., Chi0-4n/v, Kappa1), size (i.e., MolWt, ExactMW, HeavyAtomMolWt), polarity (i.e., LabuteASA, NOCount, NumHeteroatoms) and molecular complexity (BertzCT). Chico et al. [22] have previously identified that molecular weight, polar surface area and lipophilicity of CPSMs were comparatively lower than those of KDs. These differences could be partly explained by a larger proportion of nitrogen atoms in KDs. Higher molecular weight (Figure 2d) and polarity in MDKIs could result from their supernumerary oxygen atoms. The number of heteroatoms (e.g., nitrogen, oxygen and, occasionally, sulphur) directly impacted on the number of moieties capable of hydrogen bonding, well represented among KDs and MDKIs. We linked the elevated values of molecular complexity (Figure 2f) among MDKIs to the presence of many rotational bonds and highly flexible structures. Indeed, Grabowski and Schneider [35] observed that natural products and drugs contained a similar number of rotational bonds (NPs 5.2; drugs 6.7), supporting their drug-like properties. By contrast, the authors reported those from marine sources as significantly enriched with an average of 11.5 rotational bonds per molecule, suggesting a higher degree of flexibility. Consequently, most MDKIs that are depicted on the right hand of Figure 2a could be heavy, highly polar and flexible structures.

**Figure 3:**
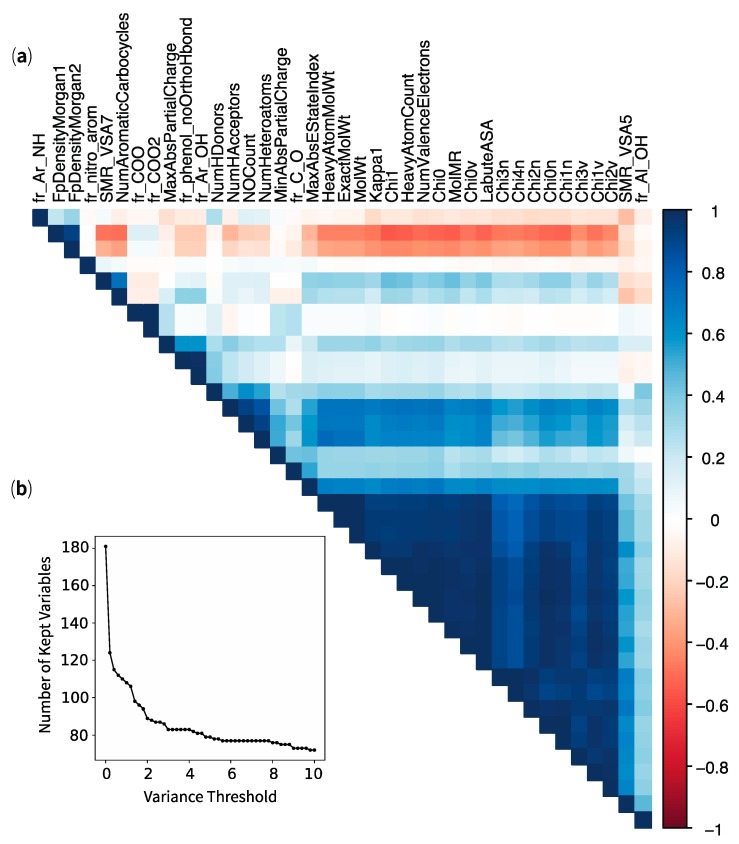
Analysis of the 200 variables with (**a**) a correlogram showing high multicollinearity (Pearson correlation rank > 0.9) between 39 variables and (**b**) a scatterplot showing the reduction of kept variables with an increasing variance threshold—19 variables have no information (not shown) and roughly 60 variables have low or no variance (threshold value close to zero). For complete correlograms with Pearson and Spearman correlation ranks, see Supplementary Materials.

Finally, many of the top-contributing physicochemical properties in Figure 2b were pointing towards the same direction and with the same intensity. In PCA, such an observation is often indicative of multicollinearity among variables. A detailed figure of these contributions is available as Supplementary Figure S2b. Therefore, we evaluated the multicollinearity between physicochemical properties using Pearson and Spearman correlation ranks. The results are presented as a correlogram (Figure 3a), whereby a strong positive correlation between variables (close to +1) appears dark blue, while a strong negative (or anti-) correlation (close to −1) appears dark red. The correlogram revealed 39 physicochemical descriptors that demonstrated high positive or negative linear correlations, with Pearson ranks over 0.9. Similar non-linear correlations were obtained with Spearman rank over 0.9 (see Supplementary Figure S3b). These results confirmed our hypothesis that many variables contributing to the principal component analysis were also highly correlated to one another. Of the 200 physicochemical descriptors, 19 were void of any physicochemical measure (NA) and were removed from the analysis. We further analysed the amount of variance among the remaining 181 variables, as illustrated in Figure 3b. About 60 properties exhibited a negligible variance threshold (close to 0) and another 40 had variance thresholds ranging from 0.5 to 10. Finally, the remaining 80 variables displayed strong variance thresholds over 10. When analysing variance threshold with 161 variables (the dataset minus the 39 highly correlated physicochemical descriptors), we noted that the decreasing trend was conserved but had merely shifted (grey line in Supplementary Figure S4). That observation suggested that all 39 variables demonstrated different variance thresholds across the range. Next, we explored the influence of these 39 variables upon different predictive models and validation.

### 2.2. Comparing Classification and Regression QSPR Models

To build our models, we picked the 332 CPSMs with in vivo logBB values to form the model set. We randomly selected 32 compounds (~10%) from this set as an external test set to evaluate the performance and validate each model independently of the optimisation process. Each model set (remaining 300 compounds) was subjected to a stratified 10-fold cross-validation—270 for training set versus 30 for testing set each time. The remaining 636 observations constituted the holdout set that included 116 CPSMs (without logBB values), 49 KDs and 471 MDKIs. In our study, we evaluated 18 predictive models – 9 regressors and 9 classifiers. Two regression models (ridge regression, Bayes ridge regression) and four classification models (logistic regression, decision tree, random forest, gradient boosting) were further optimised using variable selection and hyperparameter tuning.

Focusing on multi-label classifiers, we categorised compounds as BBB-permeable or BBB-impermeable (class membership BBB+ or BBB−) based on known in vivo logBB values. Previous studies, reviewed by Mensch and co-authors [19], reported binary classification models including recursive partitioning, k-nearest neighbour (KNN), support vector machine (SVM) or artificial neural network (ANN). In the interest of comparing these models with ours, we ensured that our study included some of the aforementioned methods, particular those built using similarly sized (~300 compounds) training sets. Of particular relevance, Li and co-workers [36] constructed binary classifiers where CPSMs were divided into BBB+ (276) and BBB− (139) groups, based on a logBB cut-off of +0.1, with 332 compounds in the training set. Thus, we set out to explore multiple binary classifiers where the two classes, namely class 1 and class 0, were separated based on a logBB cut-off of +0.1. Our results, summarised in Table 1, showed that tree-based models—decision tree (CART), random forest (RFC) and gradient boosting (GBC)—outperformed other classifiers with cross-validated (CV) accuracies of 73.4%–80.7% in the model set and 72.5%–80.0% in the external testing set. Logistic regression also exhibited strong performance in the model set, with a mean accuracy of 81.7% but a weaker performance on unseen data (67.5% in the external set). Next, we removed the 39 highly correlated variables from the dataset as it is widely considered that highly correlated variables do not improve model performances. All binary classifiers presented similar results in overall, when built from 161 or 200 variables. Both GBC and RFC remained the top performers, with mean CV accuracies of 77.4%/79.4% in the model set and 70.0%/80.0% in the external testing set. By contrast, the decision tree model showed decreased performance, with mean CV accuracies of 70.7% (model set) and 67.5% (external test set). The performance of logistic regression was improved, with a mean CV accuracy of 75.0% in the external testing set. Of note, we built and evaluated the performances of artificial neural networks binary classifiers using Python modules TensorFlow (version 1.12.0) and Scikit-learn (version 0.19.0). The results (summarised in Supplementary Table S3e,f) showed that a perceptron (single layer neural network) with 40 units would perform as well as our selected models with mean CV accuracies of 82.3% (model set) and 75.0% (external test set). Considering the complex interpretation of neural networks, we pursued our efforts towards simpler classifiers—random forest, gradient boosting and logistic regression.

Prior to further investigating these binary classifiers, we evaluated the performances of the same classifying methods when employing 2 to 5 classes. Multi-label classifiers are rare among QSPR models for BBB permeability. In 2000, Crivori et al. [37] identified logBB cut-offs using PCA and partial least squares discriminant analysis as follows: BBB− < −0.3, BBB+ > 0.0 and −0.3 ≤ BBB± ≤ 0.0. We determined the cut-off values between classes by applying k-means clustering to the logBB distribution, as presented in Supplementary Table S5. Overall, the mean CV accuracies of multi-label classifiers decreased with an increasing number of classes, both in the model set (Supplementary Table S6a) and in the external testing set (Supplementary Table S6b). The performance of binary classifiers with logBB cut-off at −0.3 (instead of +0.1) decreased by 5–6% on average. Overall, defined logBB cut-offs to generate class membership (i.e., BBB+, BBB−, BBB±) have affected the performance of our classifiers. Reverse-engineering methods could be applied to find optimal logBB cut-off(s) that maximise the performance of multi-class classifiers.

Our attempts to build regression models between logBB values and physicochemical properties led to moderate results, with 10-fold cross-validated *Q*^2^ of 0.50–0.54 and mean square errors of 0.28–0.30 in the model sets. All results are summarised in Supplementary Table S7a,b. Despite further efforts for optimisation, we were not able to improve further on these results. Of note, we have since discovered that Zhu and co-workers [21] have reported a random forest regression exhibiting good performances in training set (*R*^2^ = 0.94), testing set (*R*^2^ = 0.84) and 10-fold cross-validation (*Q*^2^ = 0.79). These results agreed with the performances of quantitative logBB models summarised by Mensch et al. [19], where *Q*^2^ ranged from 0.50 to 0.85. Our binary classifiers (with a logBB cut-off of +0.1) have demonstrated more satisfying results in terms of performance accuracies and in relation to calculated physicochemical properties from RDKit, thus, they were subjected to further optimisation.

### 2.3. Optimising Top Performing QSPR Binary Classifiers

**Figure 4:**
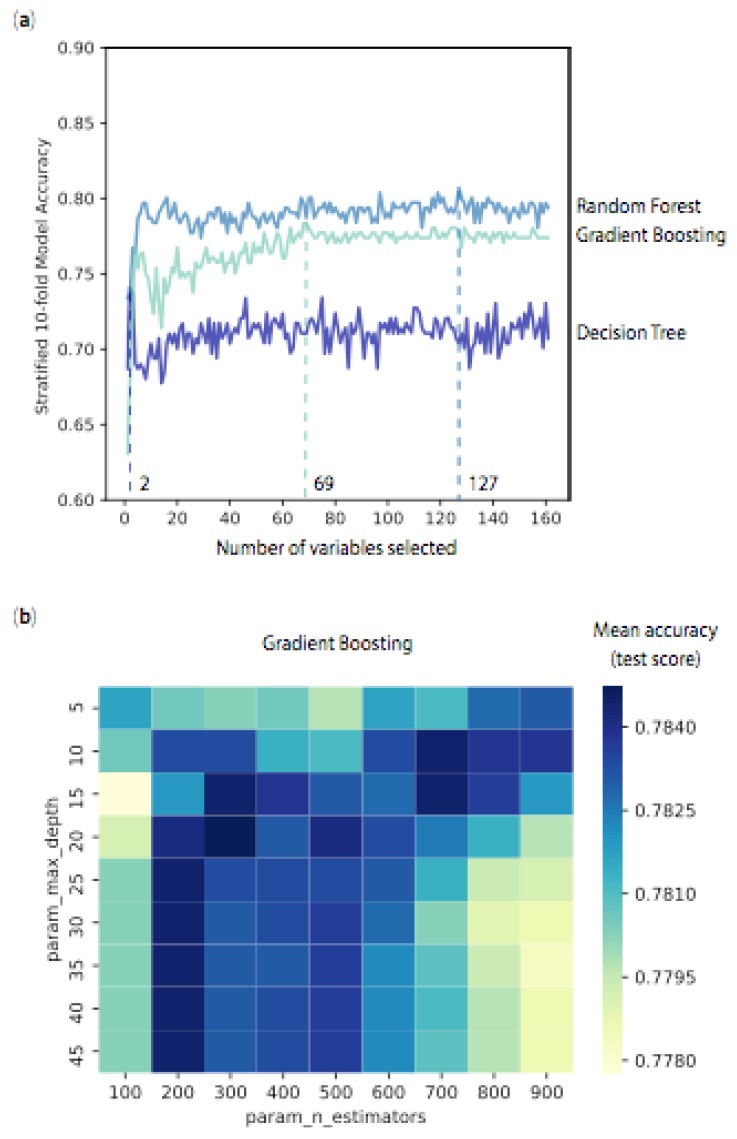
Optimisation of the 3 models—decision tree (dark blue), random forest (blue) and gradient boosting (light blue) classifiers with (**a**) dimensionality reduction using cross-validated recursive feature extraction and (**b**) tuning hyperparameters (max_depth and number of trees n_estimators) to improve performance (test mean accuracy) of gradient boosting classifier using cross-validated grid search. All illustrated hyperparameters tuning for the 3 models are presented in Supplementary Materials.

We employed two methods to optimise our models: (i) we reduced the number of variables by either multicollinearity or cross-validated recursive feature elimination (RFECV) and (ii) we tuned specific hyperparameters using a grid search. In this context, “feature” is a synonym of “variable” or “physicochemical property”. RFECV selects the best number of variables by first ranking them in order of importance in the model of interest, then, the least important variables are excluded from the entire set of variables. The procedure is repeated recursively until the desired number of variables is reached. We have summarised the performance results of RFECV (as standalone or combined to hyperparameter tuning and/or multicollinearity) in Table 2. Using the entire set of 200 variables, RFECV pruned 65 logistic regression variables, leading to mean accuracies of 82.0% in the model set and 65.6% in the external testing set (compared to 81.0% and 67.5% in the original model). Likewise, random forest binary classifier was reduced to 154 variables and gradient boosting to 160 variables. In most cases, we noted that the performances in the model set were improved by 1%–2%, while those in the external testing set were reduced by 2%–4%, which could be associated with overfitting. Similar trends occurred when building the models from 300 compounds and 161 variables. Figure 4a illustrates the application of RFECV to our decision tree, random forest and gradient boosting binary classifiers based on an original set of 161 variables. Mean accuracies were measured across the range of variables. In this figure, the desired number of variables was reached when mean accuracy in the model set was maximised. Maximum mean accuracies were reached with 2 variables for decision tree, 127 variables for random forest, and 69 variables for gradient boosting. Significantly, the accuracies flattened beyond the desired number of variables, confirming that the subsequent variables had minimal influence on the classifiers.

Each classifier has a specific set of hyperparameters that can influence the performance of the model. For example, the penalty and the cost C can be tuned for a logistic regression, while the decision tree is defined by its maximum number of variables, its depth and the number of compounds at its leaves, as summarised in Table 2 (bottom). We searched for one or more optimal hyperparameters across predefined ranges—a so-called “grid search”. These hyperparameters were considered optimal when maximum mean CV accuracies in the model set were achieved. An example of such a grid search is presented in Figure 4b, with the selection of the number of trees (param_n_estimators) and the maximum depth for each tree (param_max_depth) for gradient boosting. Changes in mean CV accuracy (internal test score from model set), from low to high values, were mirrored with a colour gradient from yellow to dark blue. Optimal hyperparameters were found when the mean CV accuracy was maximised (darkest blue). For example, gradient boosting reached maximum performance for 300 trees and a depth to 15 (branches). We have provided related illustrations of grid search for hyperparameters associated with logistic regression (Supplementary Figure S5), decision tree (Supplementary Figure S7), random forest (Supplementary Figure S6) and gradient boosting (Supplementary Figure S8) as Supplementary Figures. Initially, tuning hyperparameters of these binary classifiers had minimal effects upon random forest and gradient boosting, with mean CV accuracies (model set) of 80.0% and 78.4%, compared to 80.7% and 76.4% in the original models. Hyperparameter tuning actually had deleterious effects on the decision tree models, with mean CV accuracies lowered to 69.7% in the model set and 56.3% in the external testing set. However, when combined with removing highly correlated variables (multicollinearity), tuning hyperparameters of random forest and gradient boosting significantly improved their performances, with mean CV accuracies of 80.4% in the model set and 80.0% in the external testing set, for both models. We selected these two models as our best performers (Table 2—in bold). Following this approach, our logistic regression binary classifier outperformed other LOGREG models, with mean CV accuracies of 81.3% (model set) and 77.5% (external testing set), earning the 3rd top performing position (Table 2—in bold). Combining multicollinearity, hyperparameter tuning and RFECV had limited effects on the performances of these binary classifiers. The three optimised classifiers exhibited superior performances compared to previously reported binary classifiers [36,38].

**Figure 5:**
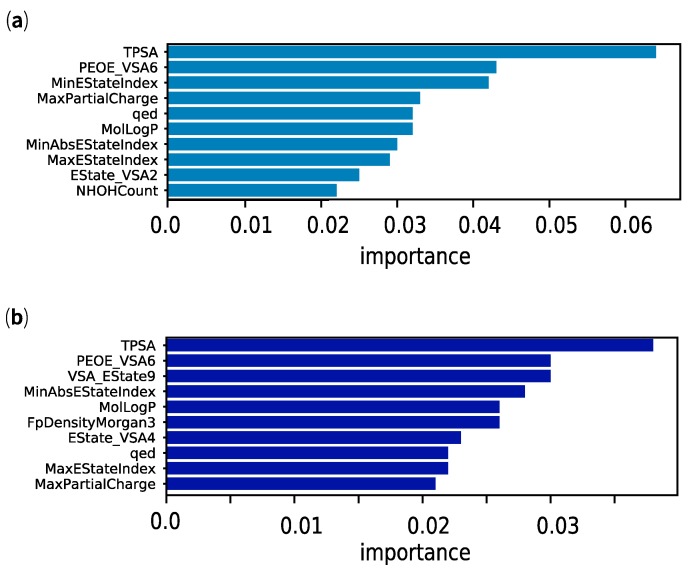
Variable importance plots for our ensemble binary classifiers (**a**) random forest and (**b**) gradient boosting.

In these ensemble models, we could extract the variables that contributed to the distinction between the two classes of CPSMs—for example, Figure 5 shows the 10 most important variables in (a) random forest and (b) gradient boosting. We noted that the hydrogen-bonding capacity of CPSMs, implicitly measured by total polar surface area (TPSA) and another molecular surface area descriptor, PEOE_VSA6, were the most significant variables in both cases. RFC and GBC also share other physicochemical properties among the most important variables, including molecular lipophilicity (MolLogP), quantitative estimate of drug-likeness (qed) [39], partial charge (MaxPartialCharge) and elements of electrotopological states (MaxEStateIndex, MinAbsEStateIndex) [40,41]. Other uniquely identified physicochemical properties of our random forest and gradient boosting models (e.g., NHOHCount) are also linked to the aforementioned properties. It is well recognised that lipophilicity and hydrogen-bonding capacity (i.e., TPSA and PEOE_VSA6) play important roles in BBB permeability [19] and, unsurprisingly, are central elements of rule-based filters for CNS drug discovery [23]. Elements of electrotopological states and partial charge are intimately connected to the electronic character of a molecule and its atoms, and are somewhat related to the hydrogen-bond capacity [40]. In Table 3, we have summarised differences in the distribution of key physicochemical properties associated with BBB permeability. We observed that CPSMs with logBB > +0.1 (CPSM+) were smaller in size, less polar (lower TPSA, lower NumHAcceptors and NumHDonors), more rigid (BertzCT, NumRotatableBonds) and more lipophilic than their non-penetrant counterparts (CPSM−). On the other hand, the quantitative estimate of drug-likeness was a less obvious property to link with BBB permeability. Developed by Bickerton and co-workers in 2012 and implemented in RDKit, qed is a composite property based on the weighted distribution of other molecular properties: molecular weight, lipophilicity, polar surface area, hydrogen-bond donors and acceptors, the number of aromatic rings, rotatable bonds and unwanted chemical functionalities [39]. In our study, CPSMs scored higher qed values (Table 3—0.7 ± 0.2) on average than KDs (0.5 ± 0.2) and MDKIs (0.4 ± 0.2). These results suggested that CPSMs exhibited more desirable physicochemical properties for orally absorbed small drugs than the two other groups. With regards to KDs and MDKIs, their physicochemical properties also appeared more extreme than CPSMs, with negative logBB values, suggesting that most compounds were unlikely to pass the BBB. After reviewing the applicability domain of our models, we have presented the promising results from our top binary classifiers applied to KDs and MDKIs. Additional and more detailed figures showing the variable importance plots from optimised random forest and gradient boosting are available as Supplementary Figures S9 and S10.

### 2.4. Identifying the Applicability Domain of our QSPR Models

The region of property space where our model predictions are considered reliable is called the domain of applicability. As we explored the BBB permeability for structurally diverse compounds (KDs, MDKIs), we were mindful that we might exceed the property space of CPSMs used in our models. Thus, we investigated the applicability domain (AD) of our binary classifiers to ensure their predictions were accurate and reliable. For over a decade, there has been a growing number of reviews and benchmark studies on methods and measures to identify AD [42,43]. Sushko et al. [44] presented the “distance to model” approach, where a distant compound would lead to an unreliable prediction. In our study, each model returned a predicted class (i.e., class 1 or BBB+ and class 0 or BBB−) and probability estimates of belonging to each class (sum to 1.0). In 2017, Klingspohn et al. [45] explored various measures for defining AD in classification models and concluded that the class probability estimates for studied classifiers were among the best AD measures. We considered our class probability estimates as excellent starting points to value, with accuracy, the class membership. Moreover, we introduced another popular AD distance-based measure known as Mahalanobis (squared) distance, for our model set and holdout set, to differentiate between reliable and unreliable results. Unlike class probability estimates, Mahalanobis distance is independent from our binary classifiers. In this study, we calculated distances for all compounds based on 138 variables (i.e., the highly correlated and low variance variables were removed).

**Figure 6:**
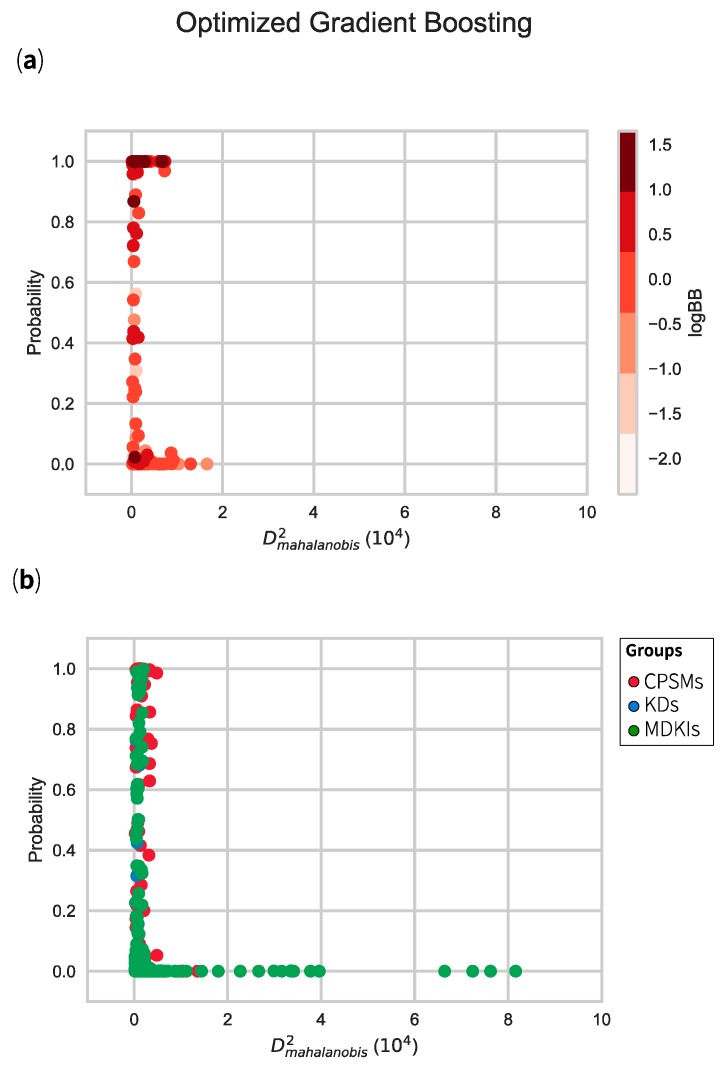
Distributions of model set (**a**) and holdout set (**b**) with class 1 probability estimates (BBB+, y-axis) and Mahalanobis squared distance (x-axis) for our optimised gradient boosting. LogBB values are displayed in a gradient of reds in the model set, while the holdout set is divided by its groups (CPSMs, KDs, MDKIs).

In Figure 6, we have illustrated the distribution of the model set (top) and the holdout set (bottom) using the class probabilities (to belong to class 1, BBB+) for optimised gradient boosting model and, their respective Mahalanobis distances. Most compounds used for training the model displayed distances less than 1 × 10^4^ framing the AD, which we referred to as *inliers*. Beyond that threshold, we observed two CPSMs as *outliers*; the macrocyclic KDs everolimus (CPSM370) and temsirolimus (CPSM433). By adding in vivo logBB values, we could evaluate their relationships with the calculated class probabilities (relating to our chosen model). In that regard, and with few exceptions, CPSMs with positive logBB values (red-dark red) exhibited high probabilities (greater than 0.7) while CPSMs with negative logBB values (white-light red) clustered at the bottom of the plot (probability < 0.3). Coincidentally, our two CPSMs outliers were reported with negative logBB values and, hence, are unlikely to pass the BBB.

We next explored how the compounds from the holdout set would distribute in the same framework. We coloured each compound by its parent group—CPSMs without logBB values (red), KDs (blue) and MDKIs (green). Overall, most compounds were inliers and displayed small Mahalanobis distances. This suggested that their class probabilities estimates were reliable. Beyond that domain, we considered that our models were extrapolating. By contrast, 19 compounds were considered as outliers, all of which were MDKIs (~3% of the holdout set). Among these outliers were sarasinosides D/E/G (MDKI34–36), hibarimicins A–D/G (MDKI105–109), dictyonamides A–B (MDKI142–143), aplidin or dehydrodidemnin B (MDKI155), glycerol derivates (MDKI175–176), oligosaccharide sulphate MdOS (MDKI215), spongistatin 1 (MDKI219), fibrosterols B–C (MDKI270–271), sesquibastadin 1 (MDKI303) and kahalalide F (MDKI309). Many of these marine natural products feature high molecular weight and high polar surface area with many heteroatoms, i.e., NOCount > 4.4 (Table 3). As discussed in Section 2.3, our models discriminated between CPSMs+ and CPSMs− using polar surface area, lipophilicity and other physicochemical descriptors associated with hydrogen-bonding capacity. Any molecule with high TPSA and high MolWeight would be classified as BBB− (with low class probability estimates). Accordingly, their class probability estimates (from optimised GBC classifier) were equal to zero. In other words, these MDKIs were very unlikely to permeate the BBB. Moreover, these outliers contain unusual peptidic or oligosaccharidic backbones, which contrasted with CPSMs. Such structural features could be associated with their “extreme” physicochemical values (beyond the property space of CPSMs) resulting in their large Mahalanobis distances. The applicability domain and predictive capacity of our models are enriched in small molecules and biased toward small molecule features. However, one cannot infer that all peptides and oligosaccharides would display large Mahalanobis distances and should be automatically considered outliers. We observed similar results in other models (Supplementary Figure S11). In the following and final section, we discuss the KDs and MDKIs that showed strong probability estimates for BBB permeation.

### 2.5. QSPR Model Results Identified Hits among KDs and MDKIs

**Figure 7:**
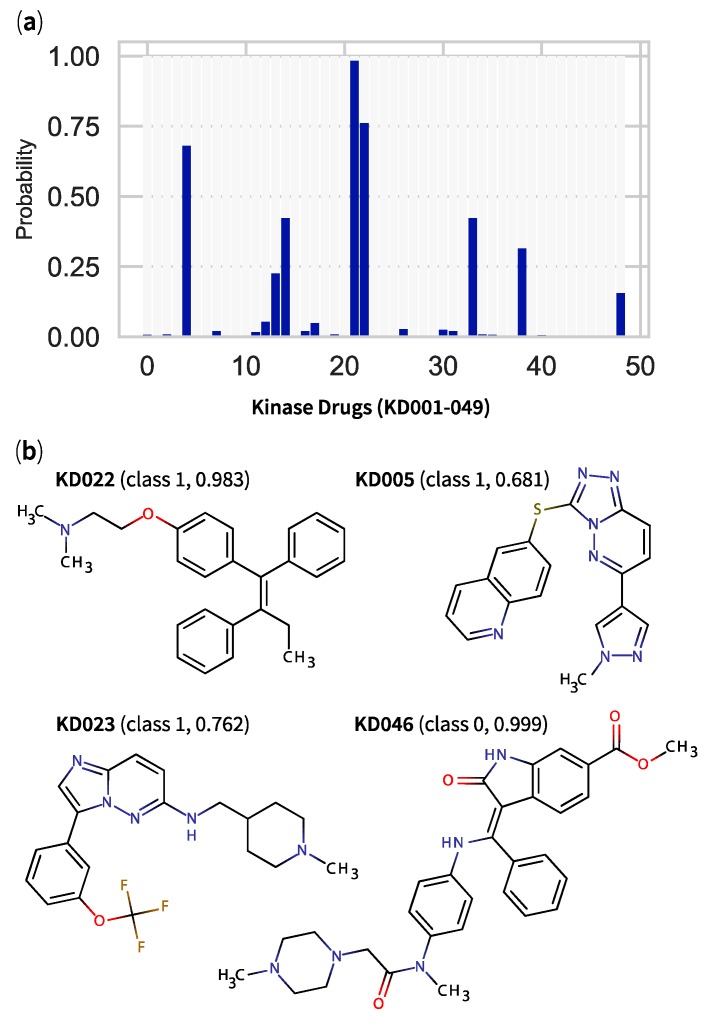
Classification results from optimised gradient boosting binary classifier applied to 49 kinase drugs (KDs) with (**a**) a histogram showing, as y-axis, the probability value for each compound to belong to class 1 (logBB > 0.1)—a compound with a probability value > 0.50 is predicted to pass the blood–brain barrier and (**b**) the structures of top candidates with their predicted classes and their respective probability values in parentheses.

**Figure 8:**
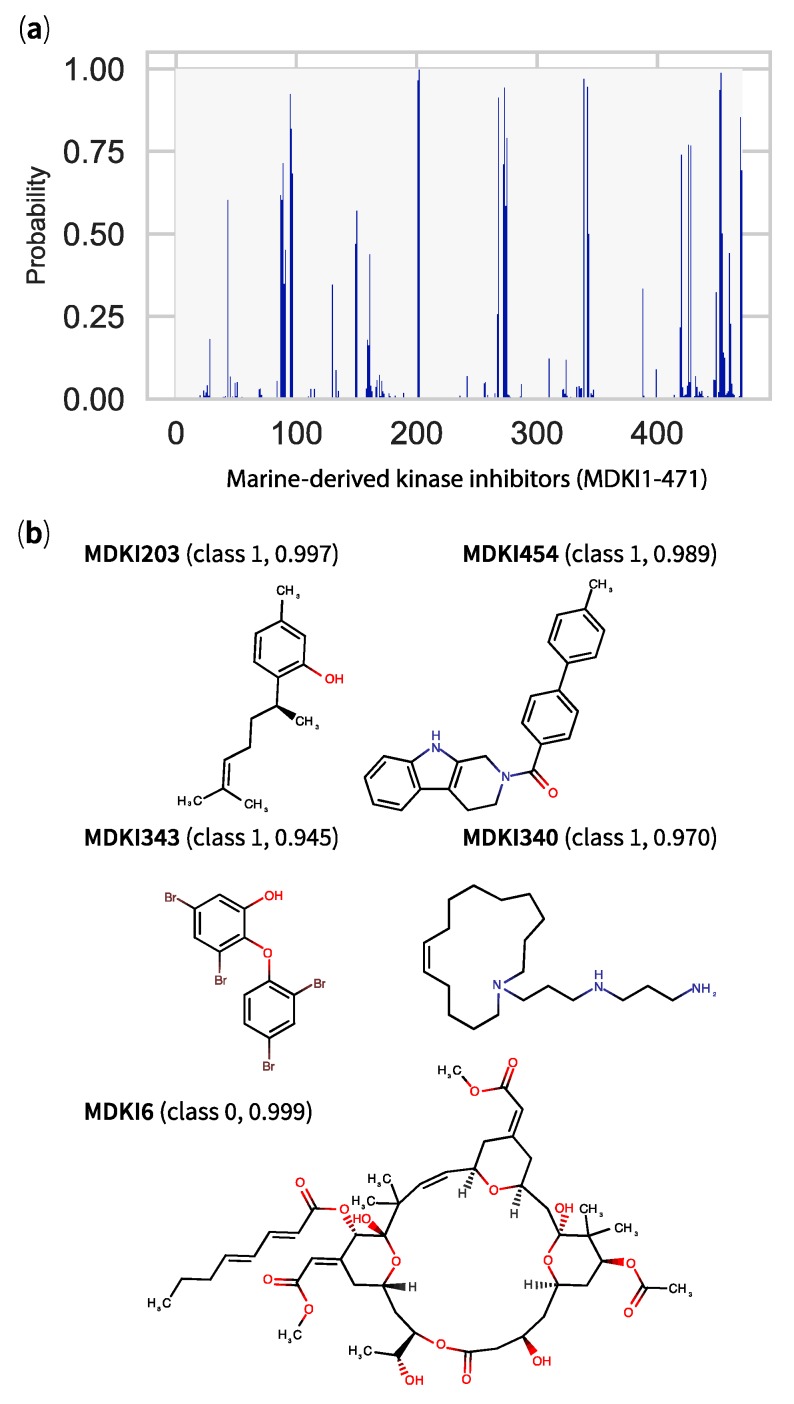
Classification results from optimised gradient boosting binary classifier applied to 471 marine-derived kinase inhibitors (MDKIs) with (**a**) a histogram showing, as y-axis, the probability value for each compound to belong to class 1 (logBB > 0.1)—a compound with a probability value > 0.50 is predicted to pass the blood–brain barrier and (**b**) the structures of top candidates with their predicted class and their respective probability values in parentheses.

After building and optimising our QSPR models, we picked two ensemble classifiers—random forest and gradient boosting—as our top performers, which gave identical mean CV accuracies of 80.4% in the model set and 80.0% in the external testing set. We also selected a third model—logistic regression—which gave mean CV accuracies of 81.3% (model set) and 77.5% (external testing set). All 3 models were tested against the holdout set consisting of 116 CPSMs (without logBB values), 49 KDs and 471 MDKIs. Each model returned a predicted class (i.e., class 1 or BBB+ and class 0 or BBB−) and probability (out of 1) of belonging to that class. The class probability estimates measured by the 3 QSPR models (RFC: blue; GBC: dark blue; LOGREG: green) for these 3 groups (CPSMs, KDs, MDKIs) are presented in Supplementary Figures S12–S14. In general, all 3 models generated comparable probability profiles. Our random forest classifier generally displayed lower probabilities than the other two models for a given compound. Here, we only discuss the results that we obtained for KDs (Figure 7) and MDKIs (Figure 8).

In agreement with the study by Chico et al. [22], most KDs demonstrated very low probability estimates (Figure 7a) implying that they were unlikely to permeate through the BBB. However, one KD—tamoxifen (KD022)—displayed promising results, appearing as the strongest hit across the 3 models (Supplementary Figure S13), with a GBC-based class probability of 0.983 (Figure 7b). Here, a hit was defined as a compound with strong class probability estimate (either >0.9 or <0.1) and short Mahalanobis distance (inlier, smaller than 1 × 10^4^). Two other KDs—SGI-1776 (KD023) and SGX-523 (KD005)—were predicted to pass the BBB with their respective moderate class probabilities of 0.762 and 0.681. Finally, nintedanib (KD046) represented the KD with the lowest probability (0.999, Figure 7b) of passing the BBB. With respect to the literature, the predicted BBB-permeable tamoxifen (KD022) was confirmed to permeate readily through the BBB [46] and the drug has demonstrated encouraging neuroprotective properties (i.e., tissue infarct and behavioural deficit prevention) in rat stroke models [47]. However, the compound has also shown an alarming risk of increased Parkinson’s disease in breast cancer patients [48,49].

From the 471 MDKIs evaluated for BBB permeability, 26 compounds (5.6%) were predicted by our gradient boosting classifier to pass the BBB, with class probabilities ranging from 0.501 to 0.997 (Figure 8a). Half (2.8%) were identified across our 3 models (Supplementary Figure S14) and 8 were categorised as recurrent hits (class probability > 0.9). Among these hits, we identified the following MDKIs (GBC-based class probability estimate in parentheses); a derivative of eudesmane (MDKI 96, 0.924), sponge-derived sesquiterpenes (+)-curcudiol (MDKI202, 0.966) and (+)-curcuphenol (MDKI203, 0.997), the alkaloid *N*,*N*-dimethyl-5-(methylthio)varacin A (MDKI269, 0.913), motuporamine C (MDKI340, 0.970), a polybrominated ether originally isolated from marine sponge *Dysidea* sp. (MDKI343, 0.945) and a synthetic analogue of fascaplysin (MDKI454, 0.989). The structures of top MDKIs hits are illustrated in Figure 8b. To this list, we could add moderate MDKIs hits: PKC inhibitors xestocyclamine A (MDKI44, 0.604) and dihydroaaptamine (MDKI421, 0.740), diterpene cembrane (2*S*,7*S*,8*S*)-sarcophytoxide (MDKI97, 0.820), nakadomarin A (MDKI273, 0.711) and the synthetic analogue of liphagal (MDKI470, 0.835). In an attempt to validate externally the use of these MDKIs in application to neurodegenerative disorders, we searched for any evidence of their BBB permeability and screened the literature for marine natural products at large. While none of the cited MDKIs appeared in our search results, we identified 3 compounds that were part of our list of MDKIs—gracilin A, bryostatin 1 (MDKI6—Figure 8b) and leucettamine B [50]. According to our best models, none of these marine compounds were predicted to pass the BBB, with GBC-based probability estimates inferior than 0.01. Gracilin A analogues were not detected in murine brain and blood matrices [51]. Synthetic derivatives of leucettamine B, leucettines, e.g., L41, displayed neuroprotective properties and corrected cognitive deficits in mouse models of Down syndrome [52,53]. Of these 3 MDKIs, only bryostatin 1 has directly been shown to permeate through the BBB. In their study, Nelson and co-authors found that bryostatin 1 was able to cross the BBB in aged rats and transgenic mice used as their neurodegenerative disease models [5]. While their findings were in contradiction with ours, it was not clear if the compound has diffused passively through BBB or via active transporter(s).

## 3. Materials and Methods

### 3.1. Data Collection

We collected a dataset of 448 CNS-penetrant small molecules (CPSMs) and 34 kinase drugs (KDs) reported by Chico and co-authors in 2009 [22]. We added 14 KDs that were approved between 2009 and 2017. Among CPSMs, 332 were reported to permeate (or not) the blood–brain barrier (BBB) with respective in vivo logBB measurements. As noted, the logBB value represents the equilibrium distribution of a compound between brain and blood (plasma) as a ratio of concentrations as shown in Equation 1, and the value is expressed between −3.0 and +2.0.

(1) $$\mathrm{logBB}=log_{10}\frac{\left[brain\right]}{\left[plasma\right]}$$

In general, compounds with logBB > 0.3–0.5 are considered to have sufficient access to the central nervous system and logBB > 1 can freely cross the BBB (i.e., passive diffusion). In comparison, logBB < 0.1 is indicative of poor or no BBB permeability. We generated the dataset of marine-derived kinase inhibitors (MDKIs) from literature search that was performed using text mining tools PubMed, Google Scholar, Scicurve and SciFinder, and combined keywords “kinase”, “inhibition”, “marine”, “natural product”. As of early 2018, the search accounted for 471 natural products published between 1982 and 2017. All 968 structures are listed with their internal codes, common names, SMILES, targeted kinases, year of discovery and references in *datasetsCompounds.csv*.

### 3.2. Data Preparation

From the original peer-reviewed articles and reviews, we reproduced all 968 compounds as two-dimensional skeletal structures including chiral centres (when known) using chemical viewer Marvin 18.3.0, 2018 ChemAxon (https://www.chemaxon.com/products/marvin, Budapest, Hungary). All 2D structural representations were cross-checked with available structures on online ChemSpider and PubChem databases before being drawn into computer-friendly SMILES strings. With the Integrated Development Environment (IDE) Jupyter Notebook version 4.3.0 (https://jupyter.org), we manipulated our chemical dataset using Python modules (pandas 0.20.3, numpy 1.15.4, matplotlib 3.0.2, scipy 0.19.1, scikit-learn 0.19.0, seaborn 0.8.0) associated with integrated Python module of the open-source Chemoinformatics software RDKit version 2017.09.3 (https://rdkit.org) (rdkit.Chem, rdkit.ML.Descriptors) for data processing and machine learning algorithms. We first imported all SMILES onto the IDE and checked that all strings were correctly depicted as skeletal structures. We then added hydrogens, removed counter-ions and neutralised charged molecules. We converted all 968 corrected SMILES into three-dimensional structures and saved them as SDF files. For each new structure, we generated 10 conformers using the conformation generation method ETKDG, implemented by Riniker and Landrum [54], and based on Cambridge Structural Database (CSD) method prior to minimise all generated conformers using MMFF94 force field. From the minimised conformers, we calculated 200 physicochemical descriptors from RDKit module Chem.Descriptors which are listed in Tables S1–S2. We stored all calculated results into a dataframe of 968 observations and 200 variables named *datasetDescrs.csv* for exploratory data analysis and model building. Prior to build models, the dataframe *datasetDescrs.csv* was pre-processed by removing duplicates, replacing missing values (NA) with zero, and normalising. Of note, only cisplatin (CPSM347) had missing values, and removing it from the dataset or replacing its missing values with zero or those of neighbouring compounds (i.e., CPSM346 or CPSM348) had no effect on the variance of variables (MinAbsPartialCharge, MaxAbsPartialCharge, MinPartialCharge, MaxPartialCharge). From the same minimised conformers, we measured molecular fingerprints (topological fingerprints, MACCS keys, atom pairs and topological torsions and Morgan circular fingerprints) in order to study molecular similarity between chemical structures. We developed an algorithm to generate and record all similarity measurements between pairs of fingerprints into symmetric matrices that were mapped into two-dimensional heatmaps for ease of interpretation. All heatmaps showed comparable results, so only MACCS keys-based heatmaps are provided as Supplementary Figure S1a,b. Some elements of exploratory data analysis, such as principal component analysis and Mahalanobis distance, were performed using R 3.3.3 (2017-03-06) and R Studio 1.0.143.

### 3.3. Model Building

We selected the 332 CPSMs with in vivo logBB values out of the 968 observations to form the model set, the dataset used to build models. The remaining 636 observations constitute the holdout set and include 116 CPSMs (without logBB values), 48 KDs and 471 MDKIs. We randomly selected 32 observations (~10%) from the model set as a test set to evaluate the performance and validate each model independently of the optimisation process. The model set (remaining 300 observations) was subjected to a stratified 10-fold cross-validation, which means the set was divided into 10 equal subsets of 30 observations (or folds) where 9 subsets were evaluated against one, 10 times. Stratified k-fold cross validation is a variant k-fold cross-validation where the folds preserve the percentage of samples of each class, which was chosen to adjust the classes imbalance. For binary classification models, the model set was divided in 2 classes based on logBB cut-offs (e.g., logBB > 0.1/class 1 or logBB ≤ 0.1/class 0), leading to a set of 111 and 189 observations, respectively. For multi-class (2–5) classifiers, the classes were set based on logBB cut-offs automatically generated using k-means clustering. Both manual and automated logBB cut-offs and the class distributions are presented in Supplementary Table S5. All classifiers were compared based on several metrics: accuracy, precision, recall, *F*_1_ score, Matthews correlation coefficient (MCC), Cohen’s kappa score κ and receiver operating characteristic area under curve (ROC AUC) value (see Supplementary Tables S3a–d, S4a–d, S6a–b). For regression models, we used all 300 observations to evaluate their performances. All regressors were compared using the following metrics: mean *R*^2^, cross-validated *Q*^2^, mean squared error, mean absolute error and explained variance (see Supplementary Table S7a,b).

In this study, we explored 18 predictive models—9 regressions and 9 classifications. The principles of all 18 statistical models are described as follows: Among the regression models, we first applied (ordinary least squares) linear regression (LINREG), illustrated by early works of Clark [55] and Platts [56], which represents the most used linear method in QSPR and describes the linear relationship between the dependent variable logBB and multiple independent physicochemical properties. We then studied three regularised linear methods—ridge regression (RIDGE), least absolute shrinkage and selection operator regression (LASSO) and elastic net regression (ELASTIC). These methods are linear methods where penalty terms, known as regularisers (e.g., L1 for LASSO, L2 for RIDGE), are added to the loss function to improve the model performance. Bayesian ridge regression (BAYESRG) estimates a probabilistic model of RIDGE as described above, where priors over α, λ, ω are estimated during the model fitness. Least-angle regression or LARS, developed by Efron et al. [57], fits a linear regression model to high-dimensional data. Support vector machine (SVR for regression, SVC for classification) is a supervised method introduced by Cortes and Vapnik [58], in 1995, that has shown good classification performance in various scenarios, including high prediction overall accuracies of 76%–84% for BBB permeability [59,60]. The concept of SVM is to first project the input data vectors (physicochemical properties) to a high-dimensional property space using the so-called kernel function prior to detect the hyperplane that separates individual classes or regression outliers with maximum margins. Finally, we evaluated two tree-based ensemble models; random forest (RFR for regression, RFC for classification) and gradient boosting (GBR, GBC). Both methods operate by constructing several decision trees (CART) in the training set and output the class or the mean prediction of individual trees. The former uses bootstrap aggregating (“bagging”) to select variables, reduce variance or avoid overfitting, while the latter, gradient boosting, applies boosting. Random forest and gradient boosting belong to a family of meta-algorithms known as ensemble methods. Other classifiers include logistic regression (LOGREG), K-nearest neighbours (KNN), linear discriminant analysis (LDA), quadratic discriminant analysis (QDA) and naïve Bayes (NB). The first uses a logistic function to model a binary output (BBB+, BBB−) while KNN matches a class membership to an observation based on a majority of its neighbours in property space. Linear and quadratic discriminant analyses assign class membership based on linear or quadratic combination of physicochemical properties. Finally, naïve Bayes applies probabilistic classification assuming all physicochemical properties are independent.

### 3.4. Variable Selection and Hyperparameter Tuning

For classifiers and regressors, in order to reduce the number of variables (dimensionality) in our dataset, we used three methods: multicollinearity between variables (Supplementary Figure S3a,b), the extraction of variables with low variance (Supplementary Figure S4) and the cross-validated recursive feature elimination (RFECV). To tune hyperparameters of our classifiers, we used a grid search with cross-validation that select hyperparameters based on model accuracy score. Examples of these searches are illustrated in Supplementary Figures S5–S8.

## 4. Conclusions

In this study, we constructed and used QSPR models to predict the ability of marine-derived kinase inhibitors (MDKIs) to pass through the blood–brain barrier (BBB), based on their physicochemical properties. We selected two ensemble binary classifiers and one logistic regression as our top performing models, which showed 80.4%–81.7% accuracy in the model set and 77.5%–80.0% accuracy in an external testing set. All our models showed excellent domains of applicability across our holdout set, with only a handful of MDKIs (~4%) and 2 CPSMs categorised as outliers. Among our best hits, we identified 3 kinase drugs (KDs; 6.1%) and 13 MDKIs (2.8%) across our models with class probability estimates over 0.9. The KD tamoxifen has been previously reported to permeate through human and rat tissues, including the BBB, providing an external experimental validation of our models’ predictions. Despite the lack of clinical evidence to validate the 13 MDKIs hits for BBB permeability, their accurate and reliable high probability estimates (within the property space of CNS-penetrant small molecules) should encourage their evaluation in models of neurodegenerative disorders. Finally, looking at the 21 outliers (2 CPSMs, 19 MDKIs) more closely, we noted these molecules were large and polar, with unusual backbones. Given their extreme physicochemical properties (Table 3), these molecules were predicted to be unlikely to pass through the BBB (very low BBB+ probability values). These observations highlight that the applicability domain and predictive capacity of our models are biased towards small molecules features and that a separate model should be developed to predict the BBB permeability of large molecules. The current lack of information about the BBB permeability of large molecules should inspire their future evaluation in models of neurodegenerative disorders.

## Figures and Tables

**Table 1:** Summary performances (accuracies) of our binary classifiers before and after removing 39 highly correlated variables (r_pearson_ > 0.9, reduced dataset). All models were built using a dataset of 300 observations and an external testing set of 32 observations under a stratified 10-fold cross-validation.

Model Name	Number Variables	Model Accuracy	External Test Accuracy
Logistic Regression	200	0.81 ± 0.06	0.68 ± 0.20
Decision Tree	200	0.73 ± 0.07	0.73 ± 0.21
Random Forest	200	0.81 ± 0.08	0.80 ± 0.22
Gradient Boosting	200	0.76 ± 0.08	0.73 ± 0.24
K-Nearest Neighbour	200	0.78 ± 0.09	0.63 ± 0.28
Linear Discriminant Analysis	200	0.70 ± 0.06	0.65 ± 0.23
Quadratic Discriminant Analysis	200	0.62 ± 0.03	0.60 ± 0.20
Naïve BAYES	200	0.59 ± 0.09	0.53 ± 0.18
Support Vector Machine	200	0.63 ± 0.01	0.68 ± 0.20
Logistic Regression	161	0.81 ± 0.05	0.75 ± 0.22
Decision Tree	161	0.71 ± 0.06	0.67 ± 0.19
Random Forest	161	0.79 ± 0.08	0.80 ± 0.22
Gradient Boosting	161	0.77 ± 0.08	0.70 ± 0.22
K-Nearest Neighbour	161	0.76 ± 0.10	0.60 ± 0.30
Linear Discriminant Analysis	161	0.75 ± 0.06	0.70 ± 0.25
Quadratic Discriminant Analysis	161	0.62 ± 0.05	0.58 ± 0.23
Naïve Bayes	161	0.56 ± 0.08	0.55 ± 0.15
Support Vector Machine	161	0.63 ± 0.01	0.75 ± 0.22

**Table 2:** Summary performances (accuracies) of our best binary classifiers after removing 39 highly correlated variables (r_pearson_ > 0.9, multicollinearity), applying recursive feature extraction and/or tuning hyperparameters. All models were built using a training set (300 observations) and an external testing set (32 observations) with stratified 10-fold cross-validation.

Model Name	Number Variables	Hyperparameters	Model Accuracy	External Test Accuracy
After Recursive Feature Elimination with Cross-Validation (RFECV)				
logistic regression	135	default	0.82	0.66
decision tree	200	default	0.75	0.69
random forest	154	default	0.82	0.78
gradient boosting	160	default	0.78	0.72
After Tuning Hyperparameters				
logistic regression ^1^	200	l1, 1	0.81 ± 0.06	0.63 ± 0.18
decision tree ^2^	200	auto, 5, 1	0.70 ± 0.05	0.56 ± 0.20
random forest ^3^	200	sqrt, 400, 5, 2	0.80 ± 0.07	0.75 ± 0.20
gradient boosting ^4^	200	log2, 200, 10, 4	0.78 ± 0.07	0.69 ± 0.20
After Tuning Hyperparameters and RFECV				
logistic regression ^1^	18	l1, 1	0.82	0.63
decision tree ^2^	156	auto, 5, 1	0.77	0.63
random forest ^3^	150	sqrt, 400, 5, 2	0.82	0.72
gradient boosting ^4^	162	log2, 200, 10, 4	0.82	0.75
After Multicollinearity and RFECV				
logistic regression	88	default	0.82	0.66
decision tree	2	default	0.70	0.63
random forest	127	default	0.79	0.78
gradient boosting	69	default	0.78	0.69
After Multicollinearity and Tuning Hyperparameters				
**logistic regression** ^1^	**161**	**l2, 10**	**0.81 ± 0.06**	**0.78 ± 0.18**
decision tree ^2^	161	auto, 6, 1	0.74 ± 0.04	0.60 ± 0.20
**random forest** ^3^	**161**	**log2, 700, 5, 2**	**0.80 ± 0.07**	**0.80 ± 0.20**
**gradient boosting** ^4^	**161**	**log2, 300, 15, 4**	**0.80 ± 0.07**	**0.80 ± 0.20**
After Multicollinearity, Tuning Hyperparameters and RFECV				
logistic regression ^1^	99	l2, 10	0.83	0.72
decision tree ^2^	85	auto, 6, 1	0.76	0.63
random forest ^3^	150	log2, 700, 5, 2	0.82	0.72
gradient boosting ^4^	161	log2, 300, 15, 4	0.80	0.78

Hyperparameters for ^1^ logistic regression {penalty, cost C}, ^2^ decision tree {max_features, max_depth, min_samples_leaf}, ^3^ random forest {max_features, n_estimators, max_depth, min_samples_leaf} and ^4^ gradient boosting {max_features, n_estimators, max_depth, min_samples_leaf} classifiers.

**Table 3:** Mean values and standard deviations of 10 physicochemical properties associated with blood–brain barrier (BBB) permeability among five groups: CNS-penetrant small molecules (CPSMs, 448), CPSMs with logBB > 0.1 (CPSM+, 127), CPSMs with logBB ≤ 0.1 (CPSM−, 205), kinase drugs (KDs, 49), marine-derived kinase inhibitors (MDKIs, 471) and Outliers (21).

Property	CPSMs	CPSM+	CPSM−	KDs	MDKIs	Outliers
MolWt	312 ± 127	296 ± 97	326 ± 129	460 ± 83	474 ± 246	1343 ± 265
TPSA	55 ± 34	36 ± 26	68 ± 28	96 ± 42	114 ± 83	399 ± 162
MolLogP	2.9 ± 1.7	3.4 ± 1.2	2.7 ± 1.9	4.2 ± 1.4	3.2 ± 2.6	1.8 ± 6.3
NumHDonors	1.2 ± 1.0	0.8 ± 0.8	1.5 ± 1.1	2.3 ± 1.9	3.0 ± 2.6	10.1 ± 5.5
NumHAcceptors	3.8 ± 2.2	3.1 ± 1.8	4.3 ± 2.0	6.4 ± 1.8	6.5 ± 5.0	23.1 ± 10.2
NOCount	4.4 ± 2.4	3.2 ± 1.9	5.2 ± 2.1	7.5 ± 2.4	7.6 ± 5.5	26.5 ± 10.6
MolMR	86 ± 36	84 ± 27	89 ± 36	123 ± 22	122 ± 59	325 ± 61
NumRotatableBonds	4.1 ± 3.0	3.5 ± 2.6	4.7 ± 3.3	5.9 ± 2.8	5.5 ± 6.9	23.1 ± 12.0
Hall–Kier Alpha	−1.9 ± 1.0	−1.7 ± 0.9	−2.1 ± 1.0	−3.4 ± 0.8	−2.6 ± 1.6	−4.2 ± 3.3
BertzCT	670 ± 367	619 ± 301	714 ± 375	1248 ± 257	1142 ± 669	2985 ± 1102
qed	0.7 ± 0.2	0.7 ± 0.2	0.6 ± 0.2	0.5 ± 0.2	0.4 ± 0.2	0.06 ± 0.03

## Supplementary Materials

The following are available online at http://www.mdpi.com/1660-3397/17/2/81/s1, Tables S1 and S2: RDKit descriptors and fragments definitions, Table S3: Performance results of binary classifiers. Table S4: Performance results of binary classifiers after RFECV and hyperparameter tuning. Table S5: Classes and logBB cut-offs. Table S6: Performance results of multi-class classifiers. Table S7: Performance results of regressors, Figure S1: Fingerprint similarity maps. Figure S2: Principal component analysis screeplot and loadings. Figure S3: Correlograms showing highly correlated variables. Figure S4: Variance threshold. Figures S5–S8: Hyperparameter tuning plots for selected QSPR models. Figures S9 and S10: Variable importance plots, Figure S11: Applicability domain of selected models. Figures S12–S14: Class probability estimates for CNS-penetrant small molecules, kinase drugs and marine-derived kinase inhibitors.

## Appendix A

Scripts for exploratory data analysis, model building, model validation and predictions. All scripts, data files and top 3 pickled models are made available on GitHub repository https://github.com/plissonf/BBB-Models under a MIT license.
