# Supplementary material for: Predicting Blood–Brain Barrier Permeability of Marine-Derived Kinase Inhibitors Using Ensemble Classifiers Reveals Potential Hits for Neurodegenerative Disorders
Source: Mar Drugs. 2019 Jan 29;17(2):81. doi: 10.3390/md17020081 (PMC6410078; doi:10.3390/md17020081)
Supplement: Supplementary file 1 [file marinedrugs-17-00081-s001.zip › marinedrugs-424632-supplementary_final_revision/marinedrugs-424632-supplementary_final_revision.pdf]

Supplementary Materials:

Predicting blood-brain barrier permeability of marine-derived kinase inhibitors using ensemble classifiers reveals potential hits for neurodegenerative disorders

Fabien Plisson[1,3]* and Andrew M. Piggott[2,3]

[1] CONACYT, Unidad de Genómica Avanzada, Laboratorio Nacional de Genómica para la Biodiversidad (Langebio), Centro de Investigación y de Estudios Avanzados del IPN, Irapuato, Guanajuato 36824, Mexico
[2] Institute for Molecular Bioscience, The University of Queensland, St. Lucia, QLD 4072, Australia
[3] Department of Molecular Sciences, Macquarie University, Sydney, NSW 2109, Australia andrew.piggott@mq.edu.au
* Correspondence: fabien.plisson@cinvestav.mx; Tel.: +52-(1)-462-166-3000; ext. 3036.

Table S1 RDKit Descriptors definitions

**Descriptor / Descriptor Family**	**Definition**	**References**
Gasteiger / Marsili Partial Charges	Marsili-Gasteiger atomic partial charges	*Tetrahedron* **36**:3219-28 (1980)
BalabanJ	Balaban's J topological index	*Chem. Phys. Lett.* **89**:399-404 (1982)
BertzCJ	Bertz CJ index	*J. Am. Chem. Soc.* **103**:3599-601 (1981)
Ipc	Information on polynomial coefficients	*J. Chem. Phys.* **67**:4517-33 (1977)
HallKierAlpha<br>Kappa1 – Kappa3<br>Chi0, Chi1<br>Chi0n – Chi4n<br>Chi0v – Chi4v	Hall and Kier shape index<br>Kappa shape indices<br>Atomic and carbon connectivity indices<br>Atomic and carbon nVal connectivity indices<br>Atomic and carbon valence connectivity indices	*Rev. Comput. Chem.* **2**:367-422 (1991)
MolLogP<br>MolMR	Molecular partition coefficient octanol/water<br>Molecular refractivity	Wildman and Crippen *JCICS* **39**:868-73 (1999)
TPSA	Total polar surface area	*J. Med. Chem.* **43**:3714-7, (2000)
LabuteASA<br>PEOE_VSA1 – PEOE_VSA14<br>SMR_VSA1 – SMR_VSA10<br>SlogP_VSA1 – SlogP_VSA12<br>EState_VSA1 – EState_VSA11<br>VSA_EState1 - VSA_EState10	Labute's apolar surface area<br>MOE-type descriptors using partial charges and surface area contributions<br>MOE-type descriptors using MR contributions and surface area contributions<br>MOE-type descriptors using LogP contributions and surface area contributions<br>MOE-type descriptors using EState indices and surface area contributions<br>MOE-type descriptors using EState indices and surface area contributions	*J. Mol. Graph. Mod.* **18**:464-77 (2000)
MQNs	Molecular quantum numbers	*ChemMedChem* **4**:1803-5 (2009)
HeavyAtomCount<br>NumHeteroatoms<br>NumRotatableBonds<br>NumValenceElectrons<br>NumAmideBonds<br>Num{ }Rings<br>Num{ }cycles<br>RingCount<br>FractionCSP3<br>NumSpiroAtoms<br>NumBridgeheadAtoms	Count of heavy atoms<br>Number of heteroatoms<br>Number of rotatable bonds<br>Number of valence electrons<br>Number of amide bonds<br>Number of aromatic, aliphatic and saturated rings<br>Number of hetero- and carbo- cycles<br>Total number of rings<br>Fraction of carbons sp3<br>Number of spiro atoms - single atom shared between rings<br>Number of bridgehead atoms - atom forming at least 2 bonds shared between rings	

https://www.rdkit.org/docs/GettingStartedInPython.html#riniker2

Table S2 RDKit fragments definitions

Fragments	Definition
NHOHCount	Number of NHs and OHs
NOCount	Number of Nitrogen and Oxygen atoms
NumHAcceptors	Number of Hydrogen Bond Acceptors
NumHDonors	Number of Hydrogen Bond Donors
fr-Al-COO	Number of aliphatic carboxylic acids
fr-Al-OH	Number of aliphatic hydroxyl groups
fr-Al-OH-noTert	Number of aliphatic hydroxyl groups excluding tert-OH
fr-ArN	Number of N functional groups attached to aromatics
fr-Ar-COO	Number of Aromatic carboxylic acids
fr-Ar-N	Number of aromatic nitrogens
fr-Ar-NH	Number of aromatic amines
fr-Ar-OH	Number of aromatic hydroxyl groups
fr-COO	Number of carboxylic acids
fr-COO2	Number of carboxylic acids
fr-C-O	Number of carbonyl
fr-C-O-noCOO	Number of carbonyl, excluding COOH
fr-C-S	Number of thiocarbonyl
fr-HOCCN	Number of C(OH)CCN-Ctert-alkyl or C(OH)CCNcyclic
fr-Imine	Number of Imines
fr-NH0	Number of tertiary amines
fr-NH1	Number of secondary amines
fr-NH2	Number of primary amines
fr-N-O	Number of hydroxylamine groups
fr-Ndealkylation1	Number of XCCNR groups
fr-Ndealkylation2	Number of tert-alicyclic amines (no heteroatoms, not quinine-like bridged N)
fr-Nhpyrrole	Number of H-pyrrole nitrogens
fr-SH	Number of thiol groups
fr-aldehyde	Number of aldehydes
fr-alkyl-carbamate	Number of alkyl carbamates
fr-alkyl-halide	Number of alkyl halides
fr-allylic-oxid	Number of allylic oxidation sites excluding steroid dienone
fr-amide	Number of amides
fr-amidine	Number of amidine groups
fr-aniline	Number of anilines
fr-aryl-methyl	Number of aryl methyl sites for hydroxylation
fr-azide	Number of azide groups
fr-azo	Number of azo groups
fr-barbitur	Number of barbiturate groups
fr-benzene	Number of benzene rings
fr-benzodiazepine	Number of benzodiazepines with no additional fused rings
fr-bicyclic	Number of bicyclic rings
fr-diazo	Number of diazo groups
fr-dihydropyridine	Number of dihydropyridines
fr-epoxide	Number of epoxide rings
fr-ester	Number of esters
fr-ether	Number of ether oxygens
fr-furan	Number of furan rings
fr-guanido	Number of guanidine groups
fr-halogen	Number of halogens

fr-hdrzine	Number of hydrazine groups
fr-hdrzone	Number of hydrazone groups
fr-imidazole	Number of imidazole rings
fr-imide	Number of imide groups
fr-isocyan	Number of isocyanates
fr-isothiocyan	Number of isothiocyanates
fr-ketone	Number of ketones
fr-ketone-Topliss	Number of ketones excluding diaryl, a,b-unsat.
fr-lactam	Number of beta lactams
fr-lactone	Number of cyclic esters (lactones)
fr-methoxy	Number of methoxy groups
fr-morpholine	Number of morpholine rings
fr-nitrile	Number of nitriles
fr-nitro	Number of nitro groups
fr-nitro-arom	Number of nitro benzene ring substituents
fr-nitro-arom-nonortho	Number of non-ortho nitro benzene ring substituents
fr-nitroso	Number of nitroso groups, excluding NO2
fr-oxazole	Number of oxazole rings
fr-oxime	Number of oxime groups
fr-para-hydroxylation	Number of para-hydroxylation sites
fr-phenol	Number of phenols
fr-phenol-noOrthoHbond	Number of phenolic OH excluding ortho intramolecular Hbond substituents
fr-phos-acid	Number of phosphoric acid groups
fr-phos-ester	Number of phosphoric ester groups
fr-piperdine	Number of piperdine rings
fr-piperzine	Number of piperzine rings
fr-priamide	Number of primary amides
fr-prisulfonamd	Number of primary sulfonamides
fr-pyridine	Number of pyridine rings
fr-quatN	Number of quarternary nitrogens
fr-sulfide	Number of thioether
fr-sulfonamd	Number of sulfonamides
fr-sulfone	Number of sulfone groups
fr-term-acetylene	Number of terminal acetylenes
fr-tetrazole	Number of tetrazole rings
fr-thiazole	Number of thiazole rings
fr-thiocyan	Number of thiocyanates
fr-thiophene	Number of thiophene rings
fr-unbrch-alkane	Number of unbranched alkanes of at least 4 members (excludes halogenated alkanes)
fr-urea	Number of urea groups

https://www.rdkit.org/docs/GettingStartedInPython.html#riniker2

Table S3a Performance results of binary classifiers with default hyperparameters. Models built on a training set of 300 observations × 200 variables under stratified 10-fold cross-validation and a logBB cut-off at 0.1 (logBB>0.1/class 1 or logBB≤0.1/class 0) leading to 2 classes of 111 and 189 observations.

Model	Parameters	Variables	Accuracy	Prec.	Recall	F_1	MCC	κ	ROC AUC
LOGREG	default	200	0.810 ± 0.059	0.81	0.81	0.81	0.58	0.58	0.78
CART	default	200	0.734 ± 0.066	0.74	0.73	0.73	0.43	0.43	0.72
RFC	default	200	0.807 ± 0.076	0.81	0.81	0.80	0.58	0.57	0.77
GBC	default	200	0.764 ± 0.077	0.76	0.76	0.76	0.48	0.48	0.73
KNN	default	200	0.781 ± 0.095	0.78	0.78	0.78	0.53	0.53	0.77
LDA	default	200	0.700 ± 0.065	0.71	0.70	0.70	0.38	0.38	0.69
QDA	default	200	0.624 ± 0.027	0.55	0.62	0.51	0.01	0.01	0.50
NB	default	200	0.589 ± 0.088	0.73	0.59	0.58	0.33	0.26	0.66
SVC	default	200	0.630 ± 0.007	0.75	0.75	0.75	0.46	0.46	0.73

Table S3b Performance results of binary classifiers with default hyperparameters. Models evaluated against an external testing set of 32 observations × 200 variables under stratified 10-fold cross-validation.

Model	Parameters	Variables	Accuracy	Prec.	Recall	F_1	MCC	κ	ROC AUC
LOGREG	default	200	0.675 ± 0.195	0.66	0.66	0.65	0.33	0.31	0.66
CART	default	200	0.725 ± 0.208	0.73	0.72	0.72	0.45	0.44	0.72
RFC	default	200	0.800 ± 0.218	0.78	0.78	0.78	0.56	0.56	0.78
GBC	default	200	0.725 ± 0.236	0.72	0.72	0.72	0.44	0.44	0.72
KNN	default	200	0.625 ± 0.279	0.63	0.62	0.62	0.25	0.25	0.63
LDA	default	200	0.650 ± 0.229	0.62	0.62	0.62	0.25	0.25	0.63
QDA	default	200	0.600 ± 0.200	0.59	0.59	0.59	0.19	0.19	0.60
NB	default	200	0.525 ± 0.175	0.53	0.53	0.53	0.06	0.06	0.53
SVC	default	200	0.675 ± 0.195	0.67	0.66	0.65	0.33	0.31	0.65

Models: LOGREG: logistic regression, CART: decision tree, RFC: random forest classifier, GBC: gradient boosting classifier, KNN: k-nearest neighbors, LDA: linear discriminant analysis, QDA: quadratic discriminant analysis, NB: naïve Bayes and SVC: support vector classifier. Metrics: Prec.:precision, F_1: harmonic average of the precision and the recall MCC: Matthews correlation coefficient, κ: Cohen's kappa score, ROC AUC: Receiver operating characteristic area under curve.

Table S3c Performance results of binary classifiers with default hyperparameters. Models built on a training set of 300 observations × 161 variables under stratified 10-fold cross-validation and a logBB cut-off at 0.1 (logBB>0.1/class 1 or logBB≤0.1/class 0) leading to 2 classes of 111 and 189 observations.

Model	Parameters	Variables	Accuracy	Prec.	Recall	F_1	MCC	κ	ROC AUC
LOGREG	default	161	0.809 ± 0.052	0.81	0.81	0.81	0.58	0.58	0.78
CART	default	161	0.707 ± 0.061	0.71	0.71	0.71	0.37	0.37	0.68
RFC	default	161	0.794 ± 0.075	0.79	0.79	0.79	0.54	0.53	0.75
GBC	default	161	0.774 ± 0.084	0.77	0.77	0.77	0.51	0.51	0.75
KNN	default	161	0.764 ± 0.104	0.76	0.76	0.76	0.49	0.49	0.75
LDA	default	161	0.747 ± 0.063	0.76	0.75	0.75	0.48	0.47	0.74
QDA	default	161	0.617 ± 0.049	0.55	0.62	0.52	0.02	0.01	0.50
NB	default	161	0.559 ± 0.084	0.72	0.56	0.54	0.30	0.22	0.63
SVC	default	161	0.630 ± 0.007	0.40	0.63	0.49	0.00	0.00	0.50

Table S3d Performance results of binary classifiers with default hyperparameters. Models evaluated against an external testing set of 32 observations × 161 variables under stratified 10-fold cross-validation.

Model	Parameters	Variables	Accuracy	Prec.	Recall	F_1	MCC	κ	ROC AUC
LOGREG	default	161	0.750 ± 0.224	0.72	0.72	0.72	0.44	0.44	0.72
CART	default	161	0.675 ± 0.195	0.66	0.66	0.66	0.31	0.31	0.66
RFC	default	161	0.800 ± 0.218	0.78	0.78	0.78	0.56	0.56	0.78
GBC	default	161	0.700 ± 0.218	0.69	0.69	0.69	0.38	0.37	0.69
KNN	default	161	0.600 ± 0.300	0.59	0.59	0.59	0.19	0.19	0.59
LDA	default	161	0.700 ± 0.245	0.69	0.69	0.69	0.38	0.38	0.69
QDA	default	161	0.575 ± 0.225	0.57	0.56	0.56	0.13	0.12	0.56
NB	default	161	0.550 ± 0.150	0.56	0.56	0.56	0.13	0.12	0.56
SVC	default	161	0.750 ± 0.224	0.72	0.72	0.72	0.44	0.44	0.72

Models: LOGREG: logistic regression, CART: decision tree, RFC: random forest classifier, GBC: gradient boosting classifier, KNN: k-nearest neighbors, LDA: linear discriminant analysis, QDA: quadratic discriminant analysis, NB: naïve Bayes and SVC: support vector classifier. Metrics: Prec.:precision, F_1: harmonic average of the precision and the recall MCC: Matthews correlation coefficient, κ: Cohen's kappa score, ROC AUC: Receiver operating characteristic area under curve.

Table S3e Performance results of artificial neural networks (ANN) binary classifiers on 50 epochs, ADAM optimizer and binary cross entropy. Models built on a training set of 300 observations × 161 variables under stratified 10-fold cross-validation and a logBB cut-off at 0.1 (logBB>0.1/class 1 or logBB≤0.1/class 0) leading to 2 classes of 111 and 189 observations.

Model	Parameters	Variables	Accuracy
ANN1	1 dense layer (100 units)	161	0.783 ± 0.083
ANN2	1 dense layer (80 units)	161	0.790 ± 0.077
ANN3	1 dense layer (40 units)	161	0.823 ± 0.065
ANN4	2 dense layers (80 and 40 units)	161	0.773 ± 0.080
ANN5	2 dense layers (80 and 40 units), 2 dropout layers	161	0.793 ± 0.068

Table S3f Performance results of artificial neural networks (ANN) binary classifiers on 50 epochs, ADAM optimizer and binary cross entropy. Models evaluated against an external testing set of 32 observations × 161 variables under stratified 10-fold cross-validation.

Model	Parameters	Variables	Accuracy
ANN1	1 dense layer (100 units)	161	0.725 ± 0.207
ANN2	1 dense layer (80 units)	161	0.750 ± 0.223
ANN3	1 dense layer (40 units)	161	0.750 ± 0.207
ANN4	2 dense layers (80 and 40 units)	161	0.775 ± 0.207
ANN5	2 dense layers (80 and 40 units), 2 dropout layers	161	0.775 ± 0.207

.

Table S4a Performance results of binary classifiers after recursive feature elimination with cross-validation (RFECV) and model hyperparameters tuning. Models were built on a training set of 300 observations × 200 variables under stratified 10-fold cross-validation and a logBB cut-off at 0.1 (logBB>0.1/class 1 or logBB≤0.1/class 0).

Model	Parameters	Variables	Accuracy	Prec.	Recall	F_1	MCC	κ	ROC AUC
After RFECV									
LOGREG	default	135	0.820	0.82	0.82	0.82	0.61	0.61	0.80
CART	default	200	0.747	0.75	0.75	0.75	0.46	0.46	0.73
RFC	default	154	0.817	0.80	0.80	0.80	0.57	0.56	0.77
GBC	default	160	0.777	0.77	0.78	0.77	0.51	0.51	0.75
LDA	default	31	0.807	0.84	0.83	0.83	0.65	0.65	0.83
After tuning hyperparameters									
LOGREG	L1, 1	200	0.810 ± 0.059	0.80	0.80	0.80	0.59	0.59	0.79
CART	auto, 5, 1	200	0.697 ± 0.045	0.71	0.69	0.69	0.41	0.39	0.68
RFC	sqrt, 400, 5, 2	200	0.800 ± 0.066	0.80	0.80	0.80	0.56	0.56	0.78
GBC	log2, 200, 10, 4	200	0.784 ± 0.068	0.76	0.76	0.76	0.52	0.52	0.76
LDA	0.01, svd	200	0.723± 0.069	0.73	0.71	0.71	0.43	0.43	0.69
After tuning hyperparameters and RFECV									
LOGREG	L1, 1	18	0.817	0.82	0.82	0.82	0.61	0.61	0.79
CART	auto, 5, 1	156	0.767	0.76	0.75	0.75	0.48	0.48	0.74
RFC	sqrt, 400, 5, 2	150	0.817	0.81	0.81	0.80	0.57	0.57	0.77
GBC	log2, 200, 10, 4	162	0.817	0.80	0.80	0.80	0.57	0.57	0.78

Models: LOGREG: logistic regression{parameters in order: penalty, cost C}, CART: decision tree {max_features, max_depth, min_samples_leaf}, RFC: random forest classifier {max_features, n_estimators, max_depth, min_samples_leaf}, GBC: gradient boosting classifier {max_features, n_estimators, max_depth, min_samples_leaf}, LDA: linear discriminant analysis {tol value, solver}. Metrics: Prec.: precision, F_1: harmonic average of the precision and the recall MCC: Matthews correlation coefficient, κ: Cohen's kappa score, ROC AUC: Receiver operating characteristic area under curve.

Table S4b Performance results of binary classifiers after RFECV and hyperparameters tuning. Models evaluated against an external testing set of 32 observations under stratified 10-fold cross-validation.

Model	Parameters	Variables	Accuracy	Prec.	Recall	F_1	MCC	κ	ROC AUC
After RFECV									
LOGREG	default	135	0.656	0.66	0.66	0.65	0.32	0.31	0.66
CART	default	200	0.688	0.69	0.69	0.69	0.38	0.38	0.69
RFC	default	154	0.781	0.78	0.78	0.78	0.57	0.56	0.77
GBC	default	160	0.719	0.72	0.72	0.72	0.44	0.44	0.72
LDA	default	31	0.469	0.47	0.47	0.47	-0.06	-0.06	0.47
After tuning hyperparameters									
LOGREG	L1, 1	200	0.625 ± 0.182	0.67	0.62	0.60	0.29	0.25	0.63
CART	auto, 5, 1	200	0.563 ± 0.201	0.57	0.56	0.56	0.13	0.13	0.56
RFC	sqrt, 400, 5, 2	200	0.750 ± 0.200	0.75	0.75	0.75	0.50	0.50	0.75
GBC	log2, 200, 10, 4	200	0.688 ± 0.203	0.69	0.69	0.69	0.38	0.38	0.69
LDA	0.01, svd	200	0.625 ± 0.231	0.67	0.62	0.60	0.29	0.25	0.63
After tuning hyperparameters and RFECV									
LOGREG	L1, 1	18	0.625	0.67	0.62	0.60	0.29	0.25	0.63
CART	auto, 5, 1	156	0.625	0.63	0.62	0.62	0.25	0.25	0.63
RFC	sqrt, 400, 5, 2	150	0.720	0.73	0.72	0.72	0.45	0.44	0.77
GBC	log2, 200, 10, 4	162	0.750	0.75	0.75	0.75	0.50	0.50	0.75

Models: LOGREG: logistic regression{parameters in order: penalty, cost C}, CART: decision tree {max_features, max_depth, min_samples_leaf}, RFC: random forest classifier {max_features, n_estimators, max_depth, min_samples_leaf}, GBC: gradient boosting classifier {max_features, n_estimators, max_depth, min_samples_leaf}, LDA: linear discriminant analysis {tol value, solver}. Metrics: Prec.: precision, F_1: harmonic average of the precision and the recall MCC: Matthews correlation coefficient, κ: Cohen's kappa score, ROC AUC: Receiver operating characteristic area under curve.

Table S4c Performance results of binary classifiers after recursive feature elimination with cross-validation (RFECV) and model hyperparameters tuning. Models built on a training set of 300 obs. × 161 var. under stratified 10-fold cross-validation and a logBB cut-off at 0.1 (logBB>0.1/class 1 or logBB≤0.1/class 0) leading to 2 classes of 111 and 189 observations.

Model	Parameters	Variables	Accuracy	Prec.	Recall	F_1	MCC	κ	ROC AUC
After RFECV									
LOGREG	default	88	0.824	0.82	0.82	0.82	0.61	0.61	0.79
CART	default	2	0.697	0.70	0.70	0.70	0.36	0.36	0.67
RFC	default	127	0.793	0.79	0.79	0.79	0.54	0.54	0.76
GBC	default	69	0.784	0.79	0.79	0.79	0.55	0.54	0.77
LDA	default	18	0.803	0.80	0.80	0.80	0.57	0.57	0.78
After tuning hyperparameters									
LOGREG	L2, 10	161	0.813 ± 0.060	0.81	0.81	0.81	0.60	0.60	0.80
CART	auto, 6, 1	161	0.739 ± 0.043	0.74	0.74	0.74	0.43	0.43	0.71
RFC	log2, 700, 5, 2	161	0.804 ± 0.066	0.81	0.80	0.79	0.57	0.55	0.76
GBC	log2, 300, 15, 4	161	0.804 ± 0.070	0.80	0.80	0.80	0.57	0.57	0.76
LDA	0.0001, lsqr	161	0.754 ± 0.068	0.76	0.75	0.76	0.49	0.49	0.75
After tuning hyperparameters and RFECV									
LOGREG	L2, 10	99	0.830	0.82	0.82	0.82	0.62	0.62	0.81
CART	auto, 6, 1	85	0.760	0.69	0.69	0.69	0.34	0.34	0.67
RFC	log2, 700, 5, 2	150	0.817	0.81	0.81	0.80	0.57	0.57	0.77
GBC	log2, 300, 15, 4	161	0.804	0.80	0.80	0.80	0.57	0.56	0.77

Models: LOGREG: logistic regression{parameters in order: penalty, cost C}, CART: decision tree {max_features, max_depth, min_samples_leaf}, RFC: random forest classifier {max_features, n_estimators, max_depth, min_samples_leaf}, GBC: gradient boosting classifier {max_features, n_estimators, max_depth, min_samples_leaf}, LDA: linear discriminant analysis {tol value, solver}. Metrics: Prec.: precision, F_1: harmonic average of the precision and the recall MCC: Matthews correlation coefficient, κ: Cohen's kappa score, ROC AUC: Receiver operating characteristic area under curve.

Table S4d Performance results of binary classifiers after RFECV and hyperparameters tuning. Models evaluated against an external testing set of 32 observations × 161 variables under stratified 10-fold cross-validation.

Model	Parameters	Variables	Accuracy	Prec.	Recall	F_1	MCC	κ	ROC AUC
After RFECV									
LOGREG	default	88	0.656	0.66	0.66	0.65	0.32	0.31	0.66
CART	default	2	0.625	0.63	0.62	0.62	0.35	0.35	0.67
RFC	default	127	0.781	0.78	0.78	0.78	0.57	0.56	0.77
GBC	default	69	0.688	0.69	0.69	0.69	0.38	0.37	0.76
LDA	default	18	0.531	0.53	0.53	0.53	0.06	0.06	0.53
After tuning hyperparameters									
LOGREG	L2, 10	161	0.775 ± 0.175	0.75	0.75	0.75	0.50	0.50	0.75
CART	auto, 6, 1	161	0.600 ± 0.200	0.59	0.59	0.59	0.19	0.19	0.59
RFC	log2, 700, 5, 2	161	0.800 ± 0.200	0.78	0.78	0.78	0.56	0.56	0.78
GBC	log2, 300, 15, 4	161	0.800 ± 0.200	0.78	0.78	0.78	0.56	0.56	0.78
LDA	0.0001, lsqr	161	0.550 ± 0.245	0.53	0.53	0.53	0.06	0.06	0.53
After tuning hyperparameters and RFECV									
LOGREG	L2, 10	99	0.719	0.72	0.72	0.72	0.44	0.44	0.72
CART	auto, 6, 1	85	0.625	0.63	0.62	0.62	0.25	0.25	0.63
RFC	log2, 700, 5, 2	150	0.720	0.73	0.72	0.72	0.45	0.44	0.77
GBC	log2, 300, 15, 4	161	0.781	0.78	0.78	0.78	0.56	0.56	0.78

Models: LOGREG: logistic regression {parameters in order: penalty, cost C}, CART: decision tree {max_features, max_depth, min_samples_leaf}, RFC: random forest classifier {max_features, n_estimators, max_depth, min_samples_leaf}, GBC: gradient boosting classifier {max_features, n_estimators, max_depth, min_samples_leaf}, LDA: linear discriminant analysis {tol value, solver}. Metrics: Prec.:precision, F_1: harmonic average of the precision and the recall MCC: Matthews correlation coefficient, κ: Cohen's kappa score, ROC AUC: Receiver operating characteristic area under curve.

Table S5 Classes and cut-offs were identified by applying k-means clustering upon logBB distribution and features from 332 observations.

Nb. classes	logBB cut-off(s)	class distribution
2	–0.3	132 : 200
3	–0.3 / 0.51	132 : 133 : 67
4	–1.13 / –0.3 / 0.51	43 : 89 : 133 : 67
5	–1.13 / –0.3 / 0.21 / 0.77	43 : 89 : 89 : 72 : 39

Table S6a Performance results of multi-class classifiers with default hyperparameters. Models built on a training set of 300 observations under stratified 10-fold cross-validation. LogBB cut-offs follow Table S5.

Model	Accuracy	Precision	Recall	*F1* score	Mcc	Ck	ROC AUC
2 classes							
LOGREG	0.752	0.75	0.75	0.75	0.48	0.47	0.73
CART	0.680	0.68	0.68	0.68	0.34	0.34	0.67
RFC	0.745	0.74	0.74	0.74	0.46	0.46	0.73
GBC	0.744	0.74	0.74	0.74	0.46	0.46	0.73
KNN	0.658	0.65	0.66	0.65	0.27	0.27	0.63
LDA	0.754	0.75	0.75	0.75	0.49	0.49	0.75
QDA	0.583	0.53	0.58	0.58	0.02	0.01	0.50
NB	0.704	0.70	0.70	0.70	0.38	0.38	0.69
SVC	0.590	0.35	0.59	0.44	0.00	0.00	0.50
3 classes							
LOGREG*	0.627	0.63	0.63	0.63	0.40	0.40	
CART	0.542	0.55	0.54	0.54	0.28	0.28	
RFC	0.636	0.64	0.64	0.63	0.41	0.41	
GBC	0.622	0.62	0.62	0.62	0.40	0.40	
KNN	0.556	0.56	0.56	0.56	0.29	0.29	
LDA	0.594	0.60	0.59	0.60	0.37	0.37	
QDA	0.420	0.42	0.42	0.42	0.09	0.09	
NB	0.392	0.50	0.39	0.39	0.19	0.16	
SVC	0.423	0.39	0.42	0.28	0.06	0.02	
4 classes							
LOGREG*	0.501	0.50	0.50	0.49	0.26	0.26	
CART	0.457	0.46	0.46	0.46	0.23	0.23	
RFC	0.526	0.53	0.53	0.50	0.30	0.29	
GBC	0.493	0.49	0.49	0.49	0.27	0.27	
KNN	0.456	0.45	0.46	0.45	0.23	0.23	
LDA	0.491	0.49	0.49	0.49	0.29	0.29	
QDA	0.346	0.42	0.44	0.43	0.06	0.05	
NB	0.329	0.46	0.33	0.28	0.20	0.16	
SVC	0.407	0.17	0.41	0.24	0.00	0.00	
5 classes							
LOGREG*	0.402	0.40	0.40	0.39	0.21	0.20	
CART	0.372	0.37	0.37	0.37	0.19	0.19	
RFC	0.426	0.44	0.43	0.41	0.24	0.24	
GBC	0.419	0.42	0.42	0.42	0.24	0.24	
KNN	0.375	0.37	0.37	0.37	0.19	0.19	
LDA	0.400	0.40	0.39	0.39	0.22	0.22	
QDA	0.277	0.25	0.28	0.26	0.04	0.04	
NB	0.319	0.38	0.32	0.27	0.21	0.19	
SVC	0.280	0.08	0.28	0.12	0.00	0.00	

* For multiclass logistic regression, all four solvers (sag/saga or lbfgs or newton-cg) displayed the same results.

Table S6b Performance results of multi-class classifiers with default hyperparameters. Models evaluated against an external testing set of 32 observations under stratified 10-fold cross-validation.

Model	Accuracy	Precision	Recall	*F1* score	Mcc	Ck	ROC AUC
2 classes							
LOGREG	0.700	0.51	0.69	0.59	–0.11	–0.05	0.48
CART	0.600	0.61	0.59	0.60	0.03	0.03	0.52
RFC	0.633	0.50	0.62	0.55	–0.20	0.16	0.44
GBC	0.600	0.61	0.59	0.60	0.03	0.03	0.52
KNN	0.625	0.63	0.62	0.62	0.25	0.25	0.63
LDA	0.792	0.77	0.78	0.78	0.44	0.44	0.72
QDA	0.625	0.68	0.62	0.64	0.19	0.18	0.60
NB	0.550	0.56	0.56	0.56	–0.08	–0.08	0.46
SVC	0.725	0.52	0.72	0.60	0.00	0.00	0.50
3 classes							
LOGREG*	0.462	0.44	0.44	0.43	0.14	0.14	
CART	0.443	0.44	0.44	0.44	0.15	0.15	
RFC	0.320	0.28	0.31	0.29	–0.05	–0.05	
GBC	0.337	0.29	0.31	0.30	–0.05	–0.05	
KNN	0.423	0.28	0.41	0.33	0.15	0.13	
LDA	0.395	0.36	0.38	0.36	0.04	0.04	
QDA	0.273	0.28	0.28	0.27	–0.07	–0.07	
NB	0.382	0.36	0.38	0.36	0.04	0.04	
SVC	0.348	0.24	0.34	0.23	–0.08	–0.05	
4 classes							
LOGREG*	0.435	0.28	0.38	0.31	0.03	0.03	
CART	0.310	0.26	0.28	0.27	–0.03	–0.03	
RFC	0.415	0.28	0.34	0.31	0.01	0.01	
GBC	0.355	0.28	0.34	0.31	0.02	0.02	
KNN	0.340	0.34	0.31	0.29	0.04	0.03	
LDA	0.415	0.32	0.34	0.32	0.04	0.04	
QDA	0.165	0.20	0.19	0.19	–0.14	–0.14	
NB	0.410	0.29	0.38	0.33	0.05	0.05	
SVC	0.385	0.22	0.34	0.23	–0.07	–0.04	
5 classes							
LOGREG*	0.470	0.19	0.31	0.23	0.03	0.03	
CART	0.330	0.18	0.22	0.20	–0.04	0.04	
RFC	0.470	0.23	0.31	0.27	0.07	0.07	
GBC	0.470	0.24	0.31	0.26	0.05	0.05	
KNN	0.420	0.18	0.28	0.21	0.05	0.05	
LDA	0.600	0.51	0.47	0.48	0.30	0.30	
QDA	0.060	0.08	0.09	0.08	–0.17	–0.17	
NB	0.490	0.33	0.34	0.32	0.13	0.13	
SVC	0.520	0.12	0.34	0.18	0.00	0.00	

* For multiclass logistic regression, all four solvers (sag/saga or lbfgs or newton-cg) displayed the same results.

Table S7a Performance results of regressors with default or optimized hyperparameters. Models built on a training set of 300 observations under 10-fold cross-validation.

Model	Parameters	Nb. Variables	Q^2	MSE	MAE	EV
LINREG	default	200	0.08	0.66	0.58	0.11
RIDGE	default	200	0.51	0.30	0.43	0.51
LASSO	default	200	–0.01	0.61	0.62	–0.01
ELASTIC	default	200	–0.01	0.61	0.62	–0.01
LARS	default	200	–0.01	0.61	0.62	–0.01
RFR	default	200	0.43	0.34	0.43	0.43
GBR	default	200	0.47	0.32	0.43	0.47
SVR	default	200	0.18	0.50	0.55	0.19
BAYESRG	default	200	0.50	0.30	0.43	0.50
After Recursive Feature Elimination with Cross-Validation (RFECV)						
RIDGE	default	150	0.51	0.29	0.41	0.52
BAYESRG	default	126	0.50	0.30	0.43	0.51
After tuning hyperparameters						
RIDGE	1, sparse_cg	200	0.52	0.28	0.41	0.52
BAYESRG	1e-6, 10, 10, 10	200	0.51	0.28	0.43	0.51
After RFECV and tuning hyperparameters						
RIDGE	1, sparse_cg	150	0.53	0.28	0.42	0.53
BAYESRG	1e-6, 10, 10, 10	126	0.54	0.28	0.41	0.54

Models: LINREG: ordinary least squares linear, RIDGE: ridge regression {alpha, solver}, LASSO: least absolute shrinkage and selection operator regression, ELASTIC: elastic net regression LARS: least-angle regression, RFR: random forest regression, GBR: gradient boosting regression, SVR: support vector regression, BAYESRG: Bayesian ridge regression {alpha1, alpha2, lambda1, lambda2}. Metrics: Q^2, MSE: mean square error, MAE: mean absolute error and EV: explained variance.

Table S7b Performance results of regressors with default or optimized hyperparameters. Models evaluated against an external testing set of 32 observations under 10-fold cross-validation.

Model	Parameters	Nb. Variables	Q^2	MSE	MAE	EV
LINREG	default	200	0.21	0.57	0.58	0.21
RIDGE	default	200	0.15	0.60	0.60	0.16
LASSO	default	200	–0.07	0.77	0.72	–0.07
ELASTIC	default	200	–0.07	0.77	0.72	–0.07
LARS	default	200	–0.07	0.77	0.72	–0.07
RFR	default	200	–0.09	0.78	0.70	–0.09
GBR	default	200	–0.37	0.98	0.83	–0.36
SVR	default	200	–0.08	0.77	0.73	–0.08
BAYESRG	default	200	0.00	0.72	0.68	0.02
After Recursive Feature Elimination with Cross-Validation (RFECV)						
RIDGE	default	150	0.15	0.60	0.60	0.16
BAYESRG	default	126	0.00	0.72	0.68	0.02
After tuning hyperparameters						
RIDGE	1, sparse_cg	200	0.16	0.60	0.59	0.16
BAYESRG	1e-6, 10, 10, 10	200	0.11	0.64	0.62	0.11
After RFECV and tuning hyperparameters						
RIDGE	1, sparse_cg	150	0.18	0.59	0.59	0.18
BAYESRG	1e-6, 10, 10, 10	126	0.13	0.62	0.62	0.13

Models: LINREG: ordinary least squares linear, RIDGE: ridge regression {alpha, solver}, LASSO: least absolute shrinkage and selection operator regression, ELASTIC: elastic net regression LARS: least-angle regression, RFR: random forest regression, GBR: gradient boosting regression, SVR: support vector regression, BAYESRG: Bayesian ridge regression {alpha1, alpha2, lambda1, lambda2}. Metrics: Q^2, MSE: mean square error, MAE: mean absolute error and EV: explained variance.

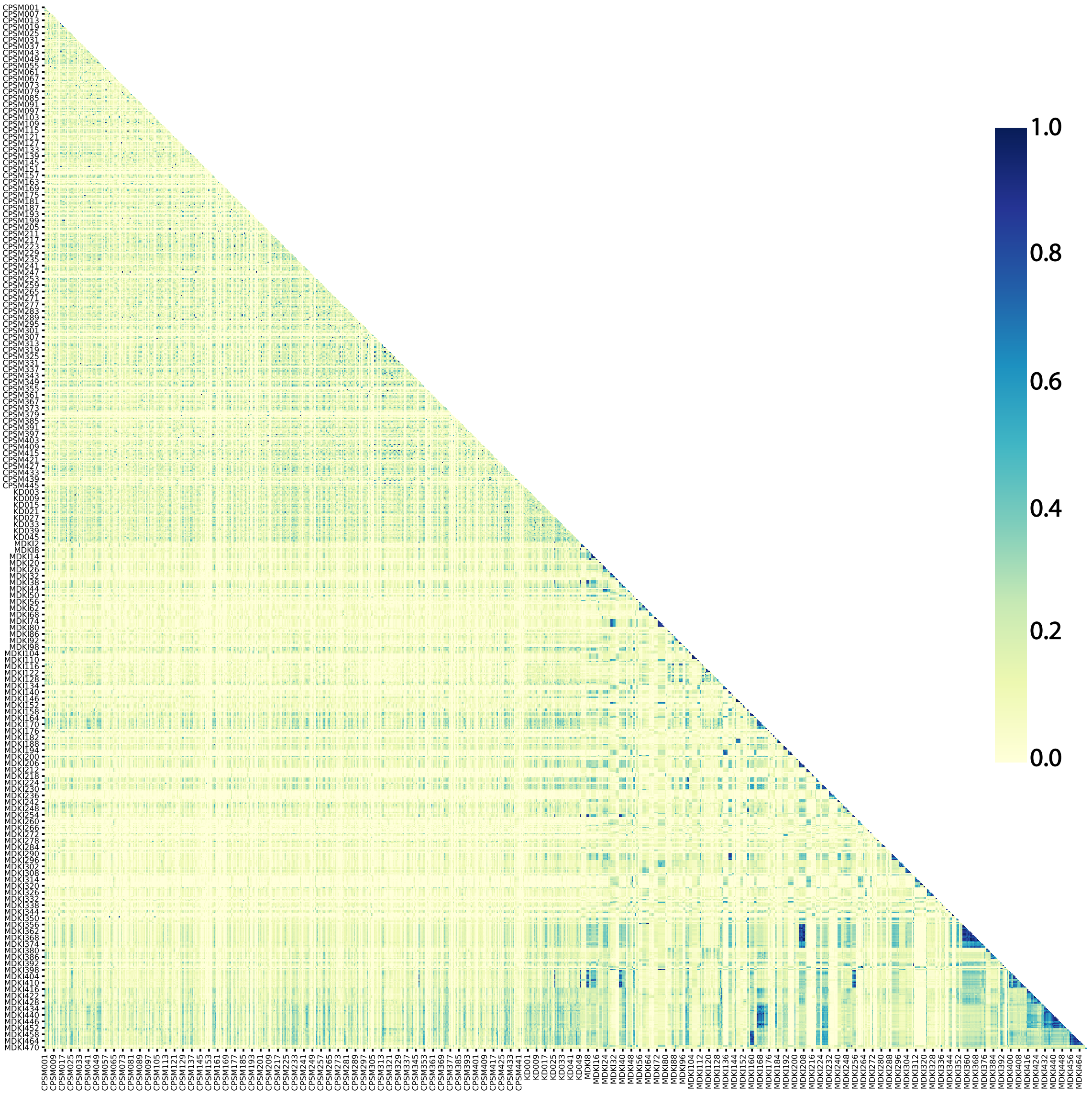

Figure S1a. Fingerprint similarity matrix using public MACCS structural keys between the 968 structures grouped as CNS-penetrant small molecules (CPSMs), kinase drugs (KDs) and marine-derived kinase inhibitors (MDKIs). The maximum similarity is observed at a value of 1.0 (dark blue).

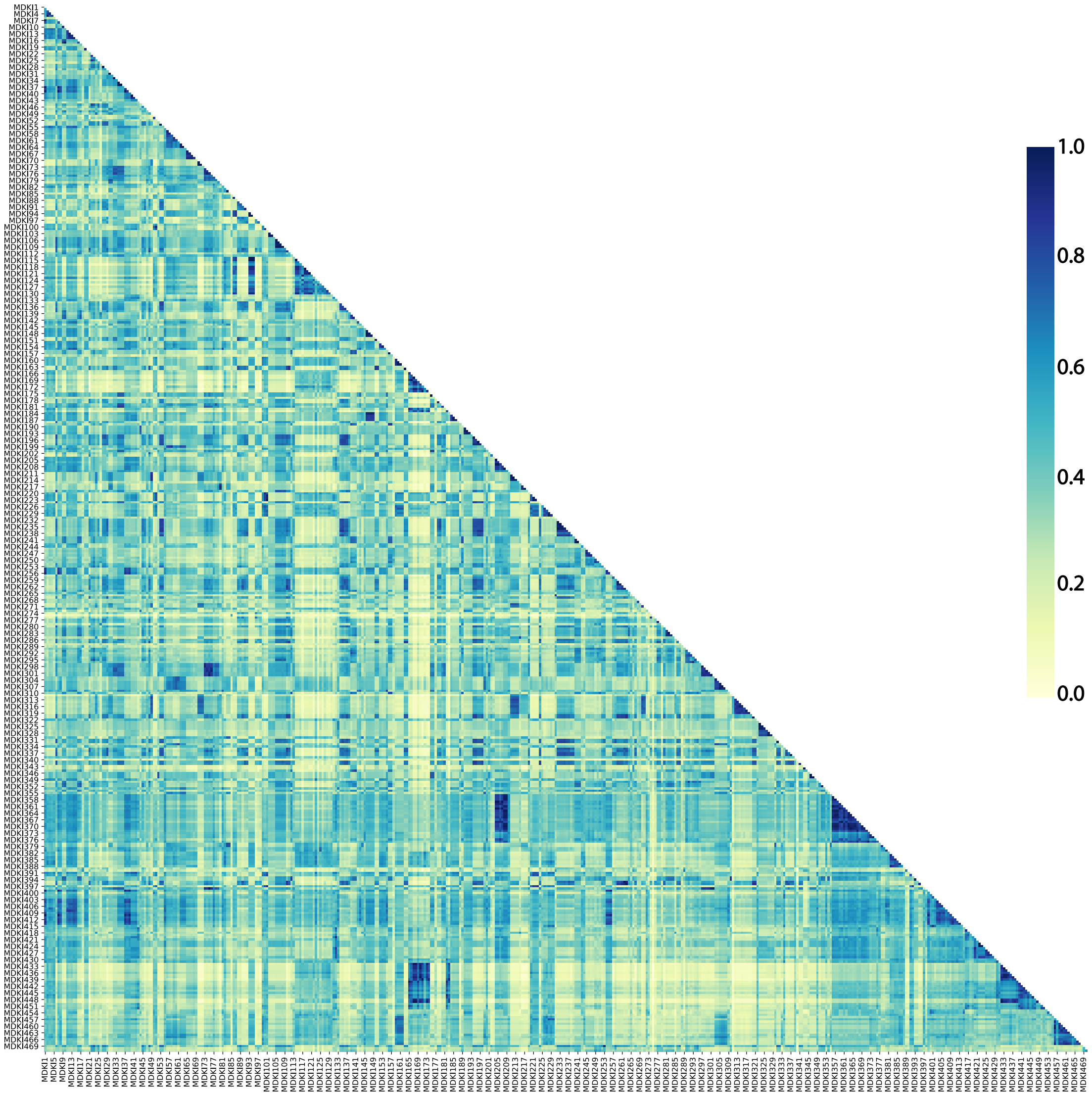

Figure S1b. Fingerprint similarity matrix using public MACCS structural keys of 471 marine-derived kinase inhibitors (MDKIs). The maximum similarity is observed at a value of 1.0 (dark blue).

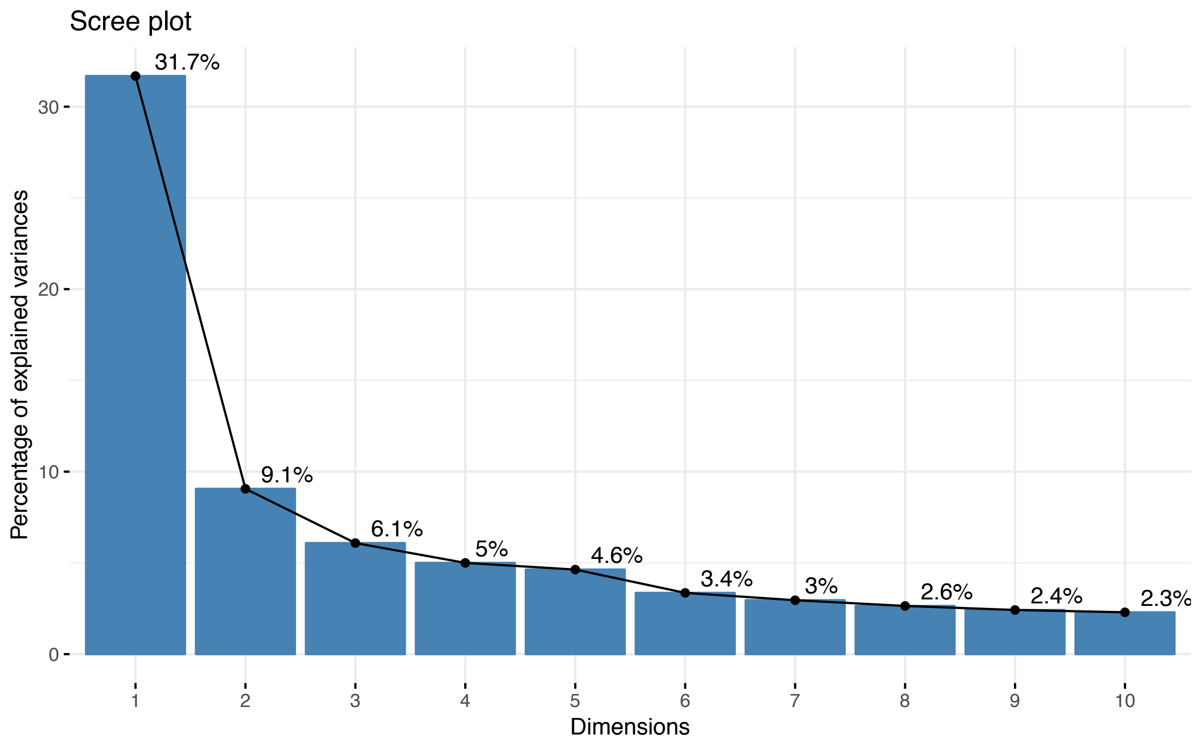

Figure S2a. Scree plot showing the first 10 principal components (dimensions) and the respective percentage of explained variance.

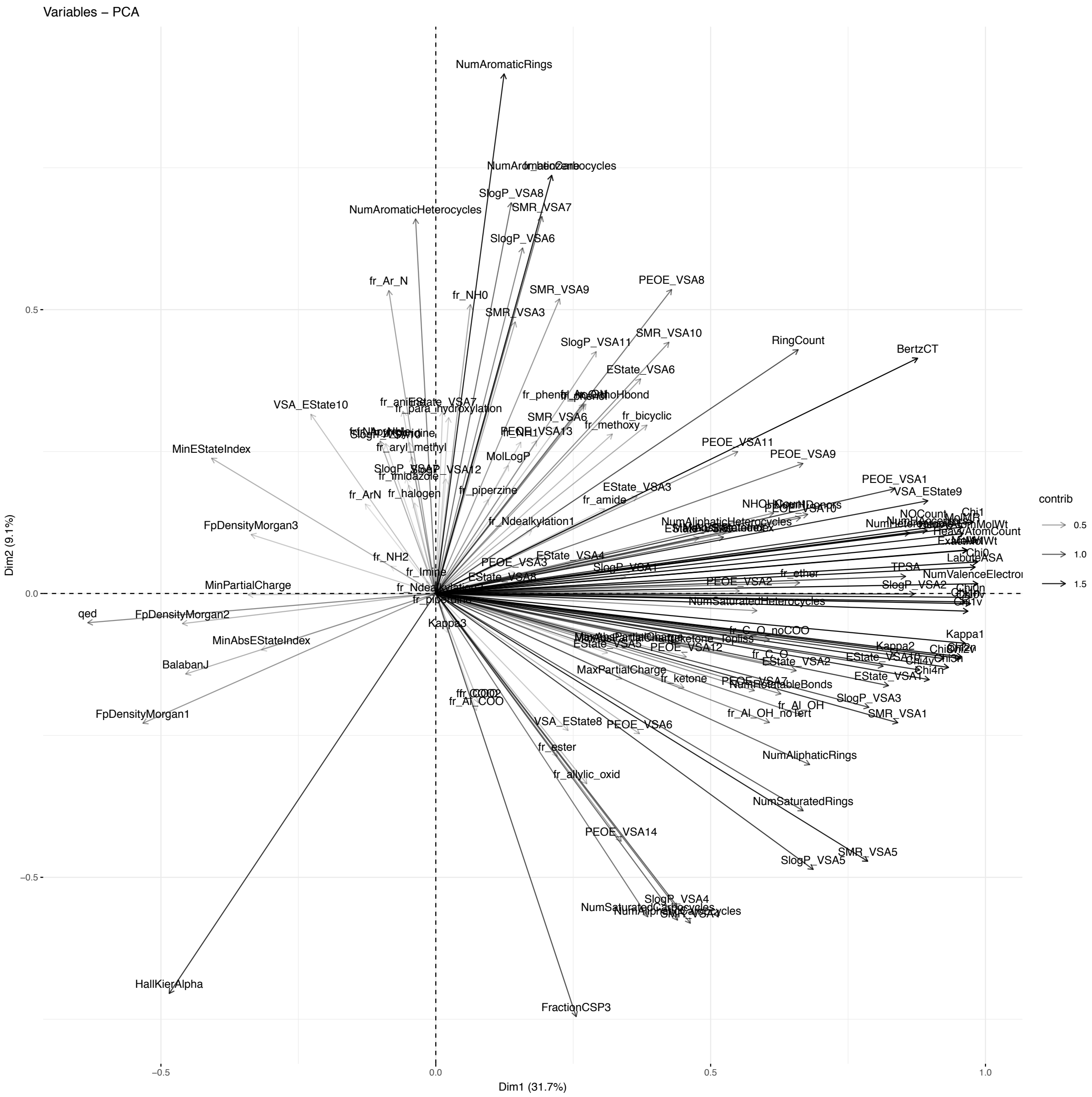

Figure S2b. Distribution of CNS-penetrant small molecules (CPSMs, red), kinase drugs (KDs, blue) and marine-derived kinase inhibitors (MDKIs, green) along the first two principal components (PCA) with loadings representing all contributing variables.

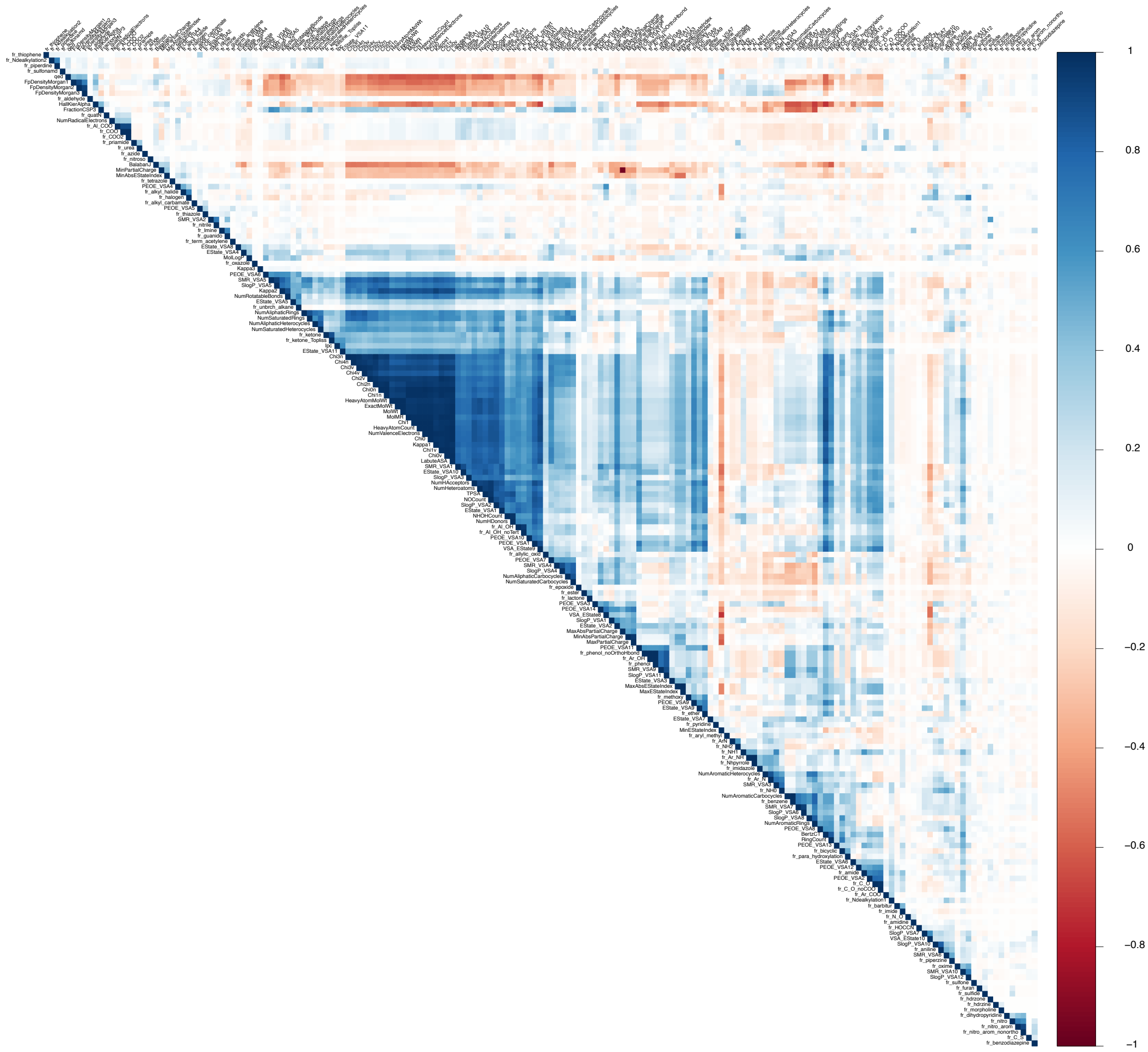

Figure S3a. Correlogram using Pearson rank showing linear correlations between the 181 variables (19 variables with no information).

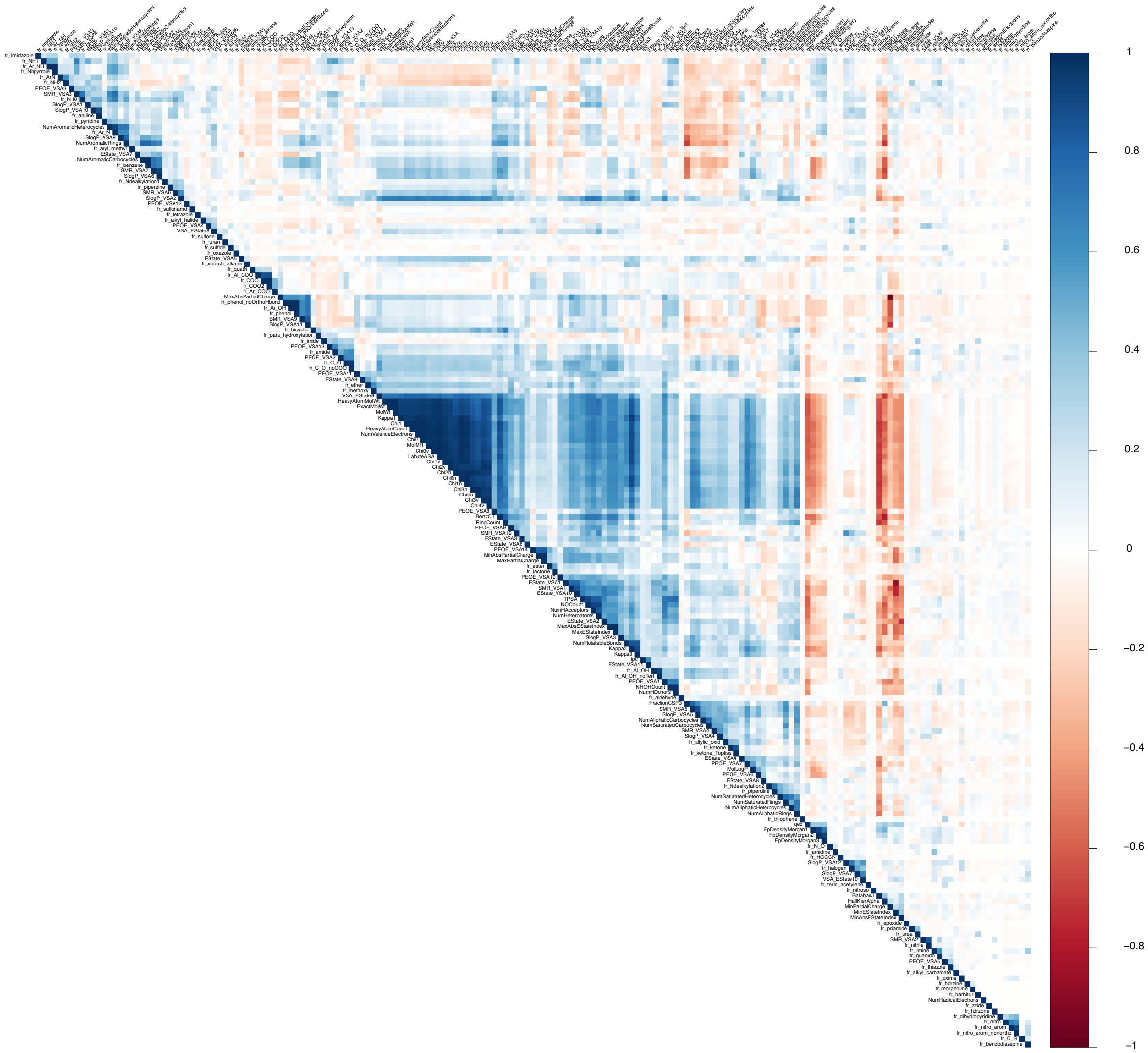

Figure S3b. Correlogram using Spearman rank showing non-linear correlations between the 181 variables (19 variables with no information).

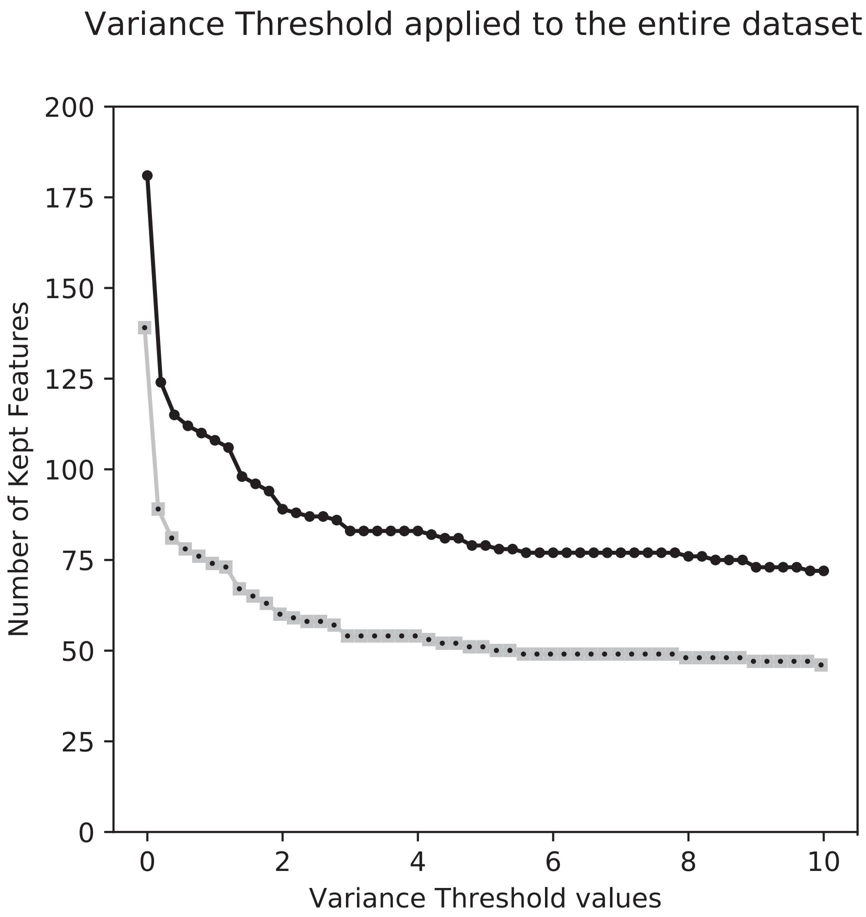

Figure S4. Scatterplot showing the reduction of kept variables with an increasing variance threshold for entire dataset (black - 200 variables) and reduced dataset (grey - 161 variables, 39 highly correlated variables were removed). In both cases, 19 variables have no information thus no variance to be shown.

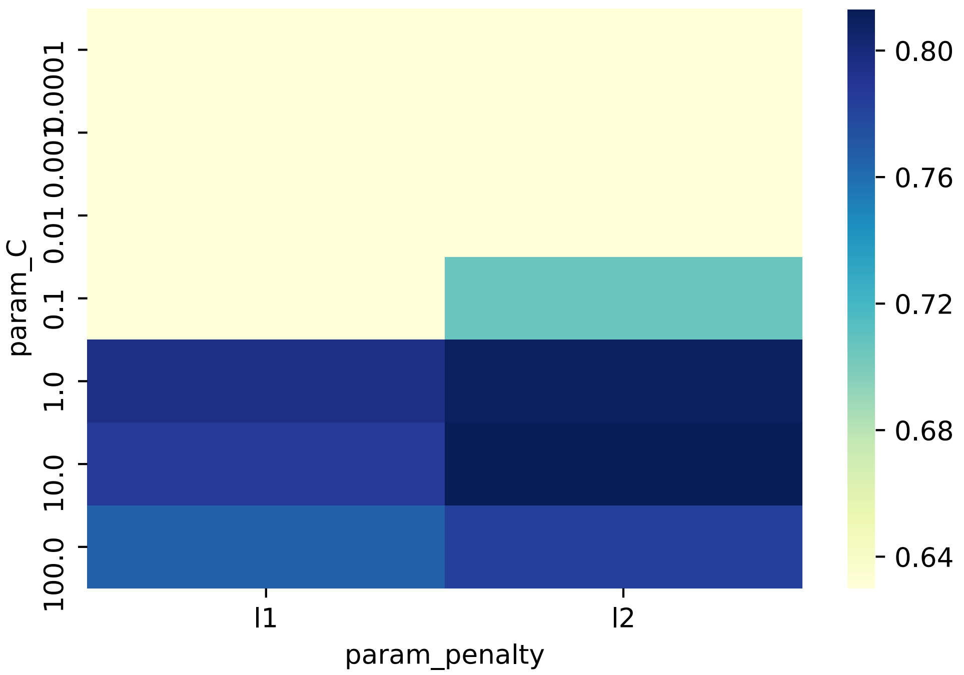

Figure S5. Hyperparameters tuning for logistic regression binary classifier

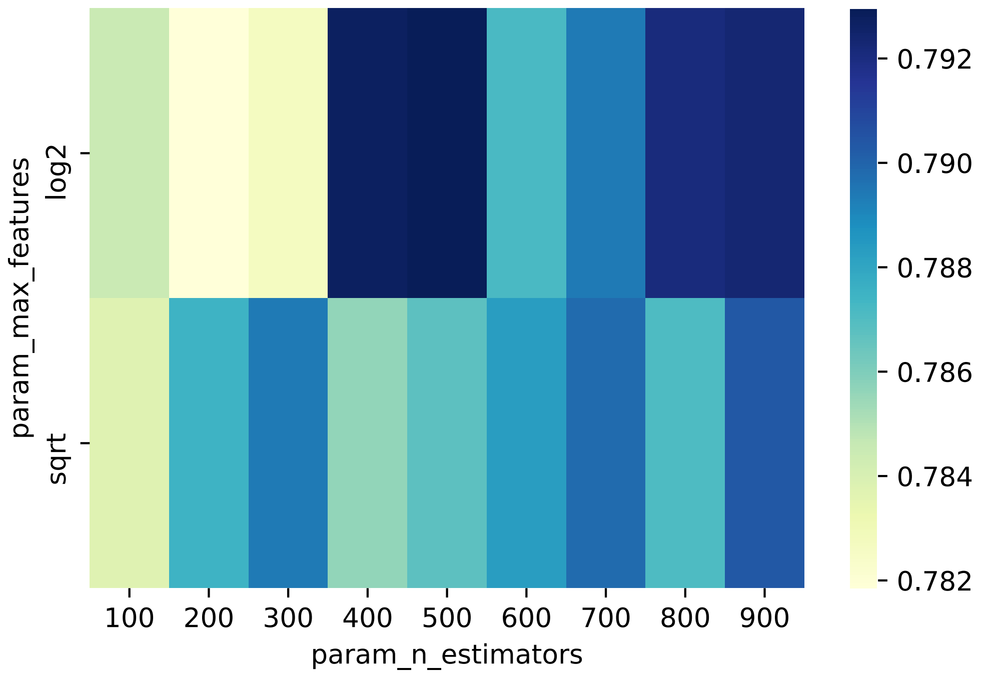

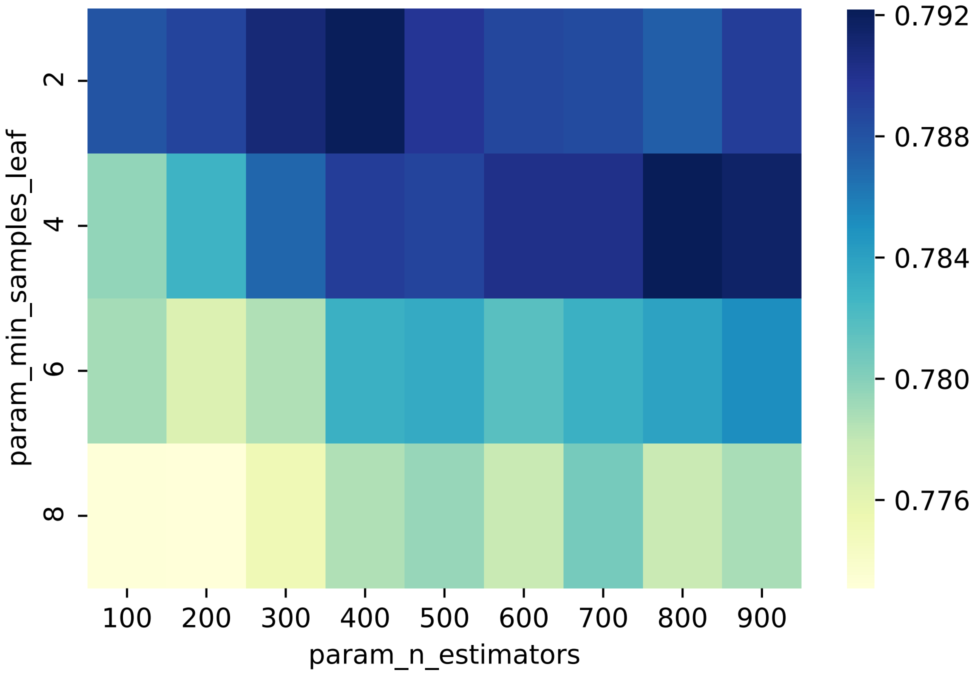

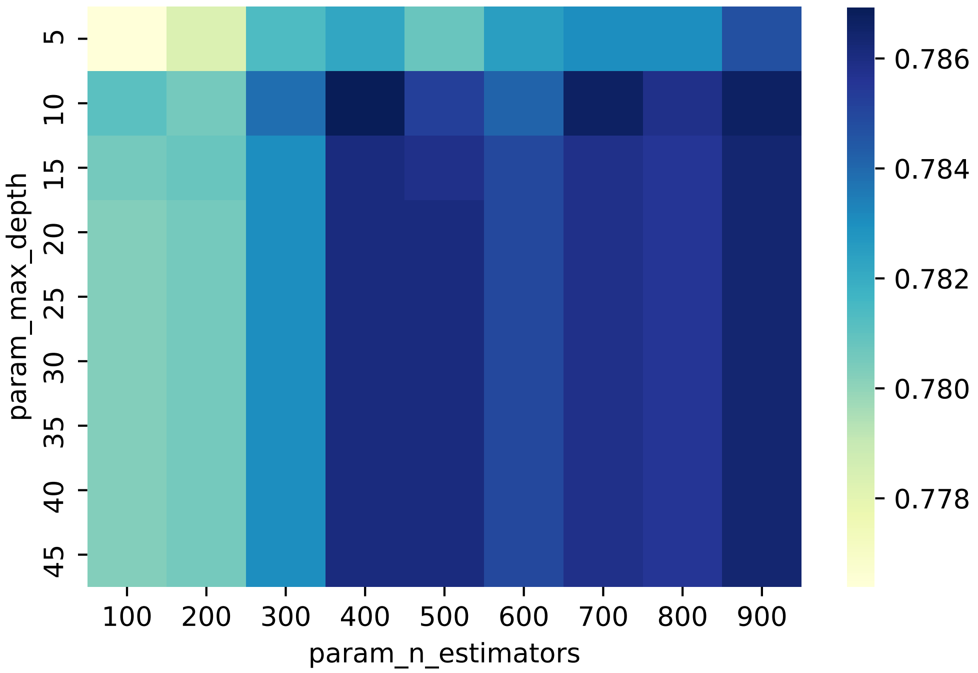

Figure S6. Hyperparameters tuning for random forest binary classifier

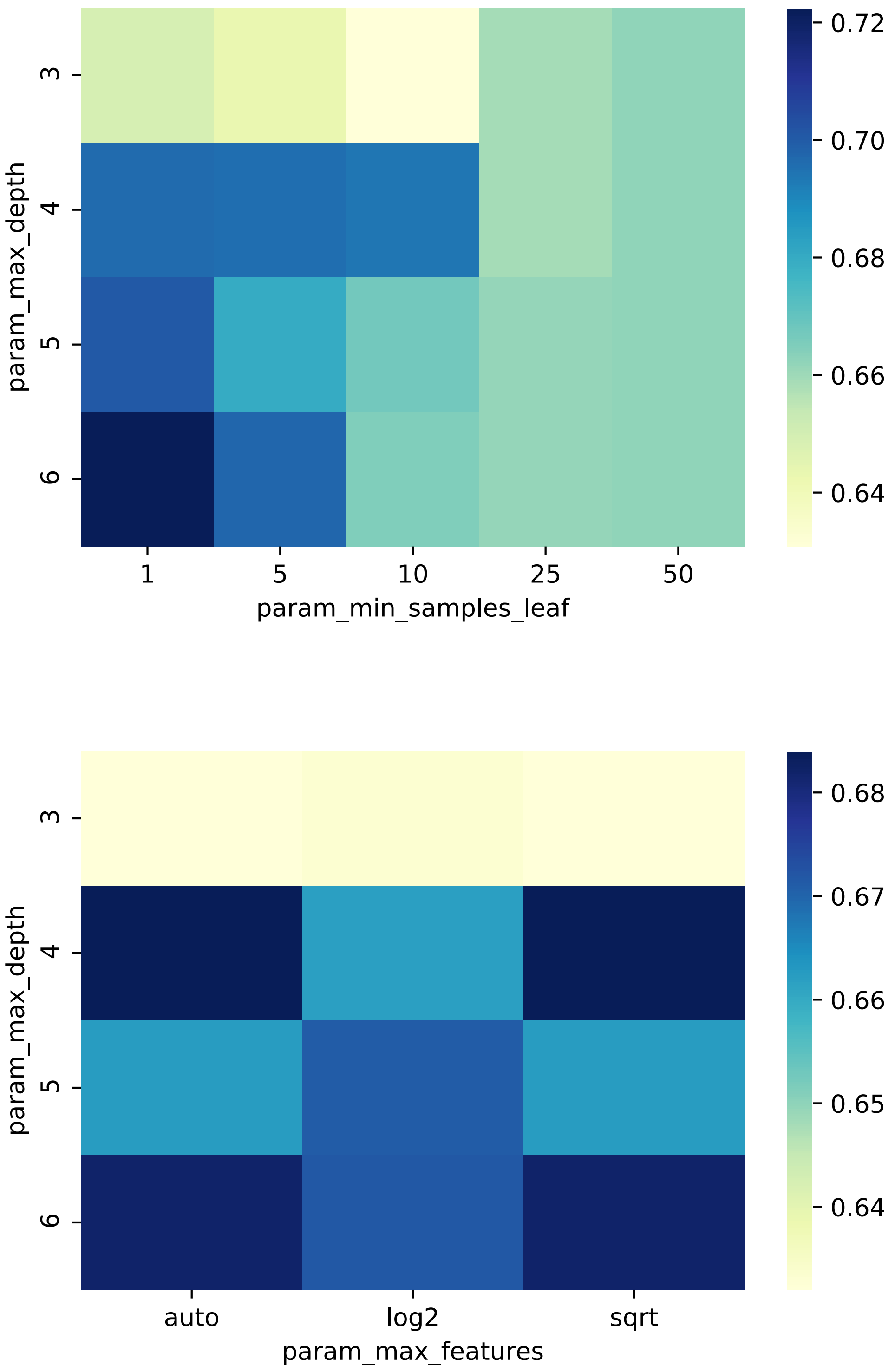

Figure S7. Hyperparameters tuning for decision tree binary classifier

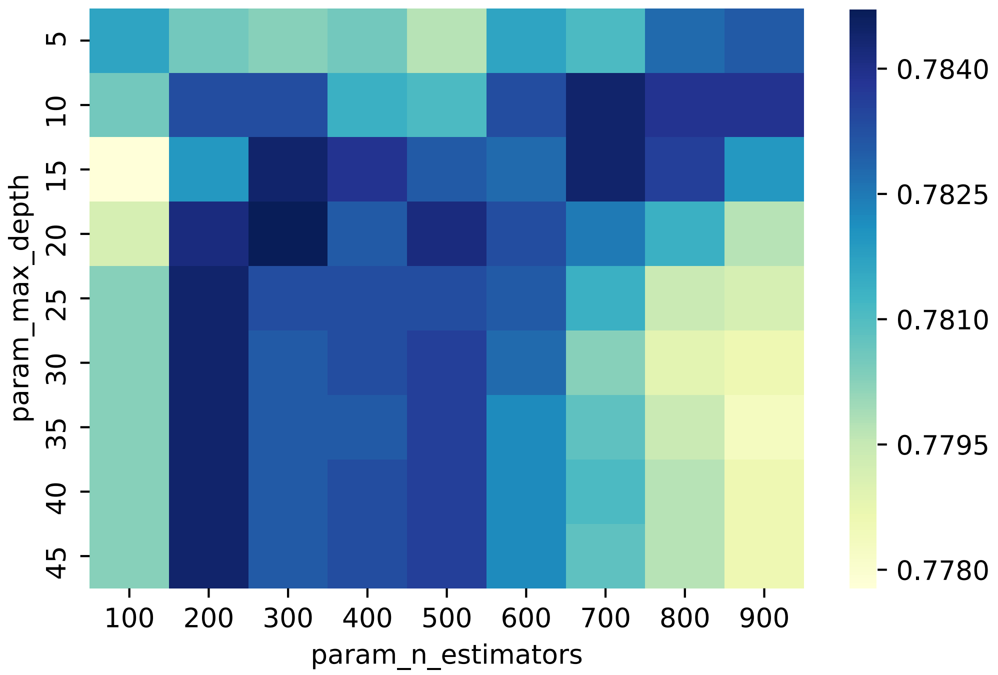
param_max_depth
5
10
15
20
25
30
35
40
45
100
200
300
400
500
600
700
800
900
param_n_estimators
0.7840
0.7825
0.7810
0.7795
0.7780

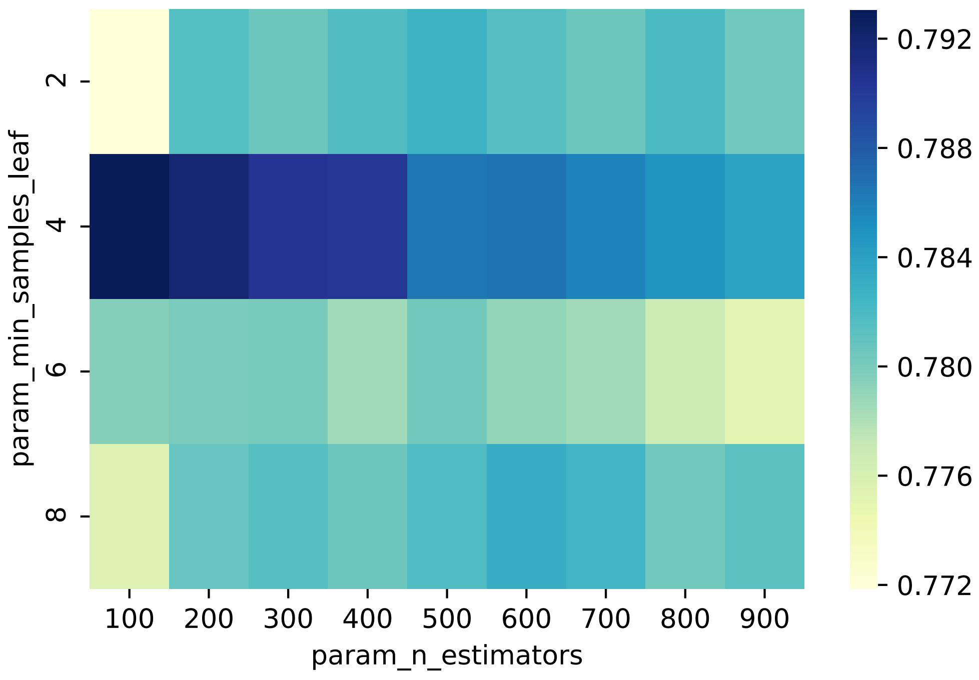
param_min_samples_leaf
2
4
6
8
100
200
300
400
500
600
700
800
900
param_n_estimators
0.792
0.788
0.784
0.780
0.776
0.772

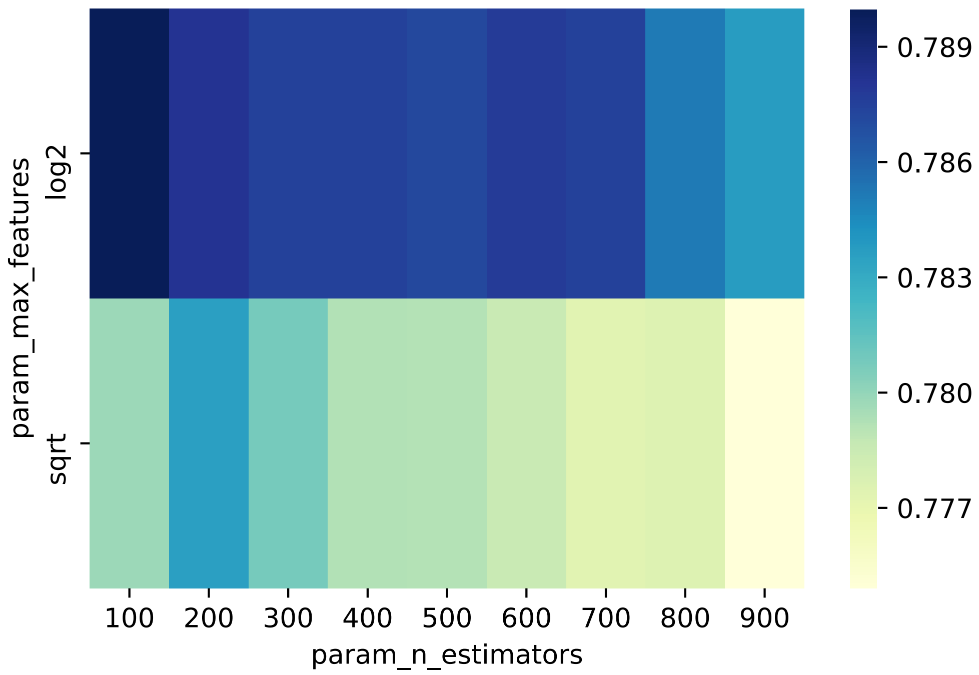

Figure S8. Hyperparameters tuning for gradient boosting binary classifier

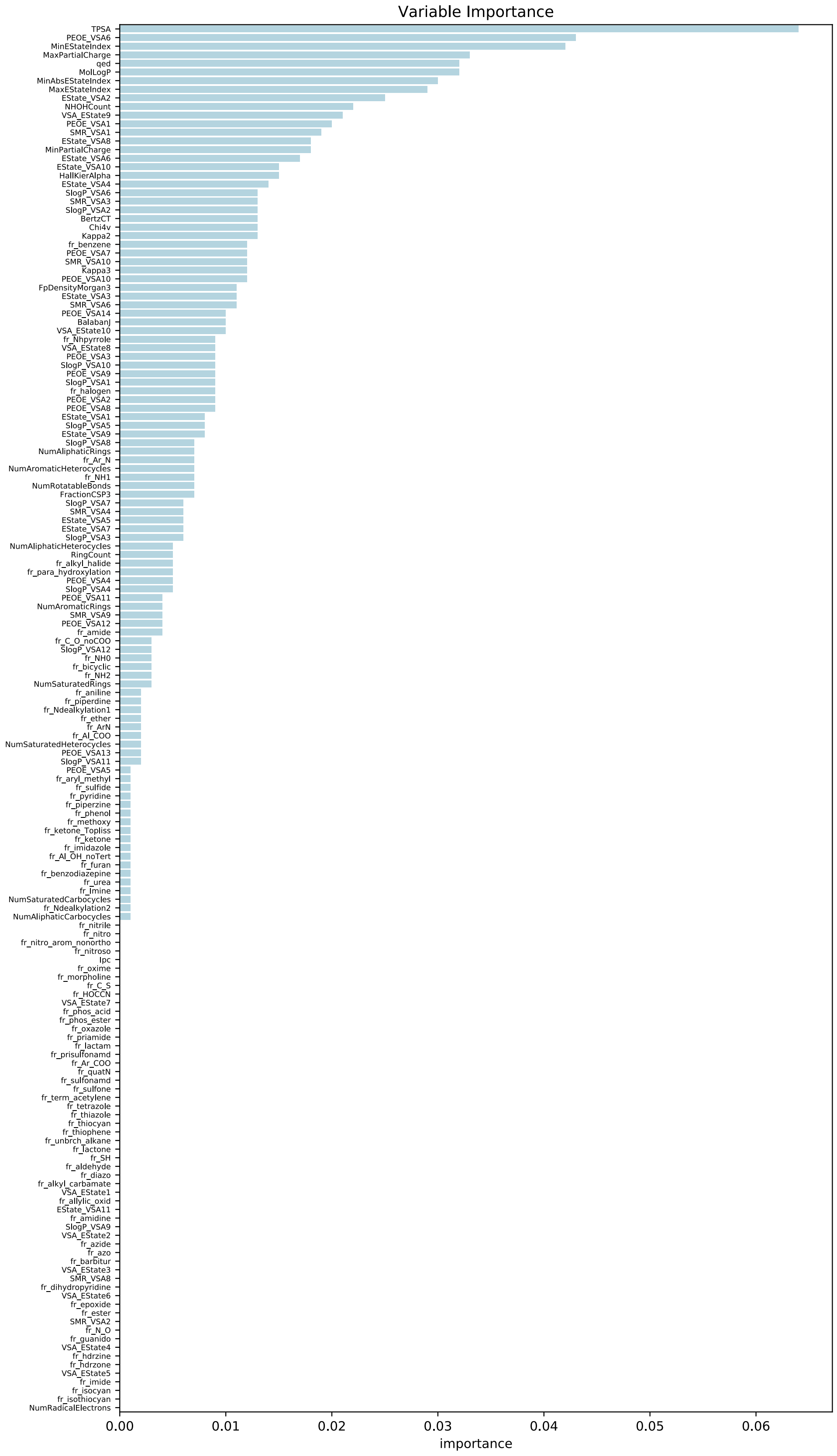

Figure S9. Variables importances for optimized random forest classifier.

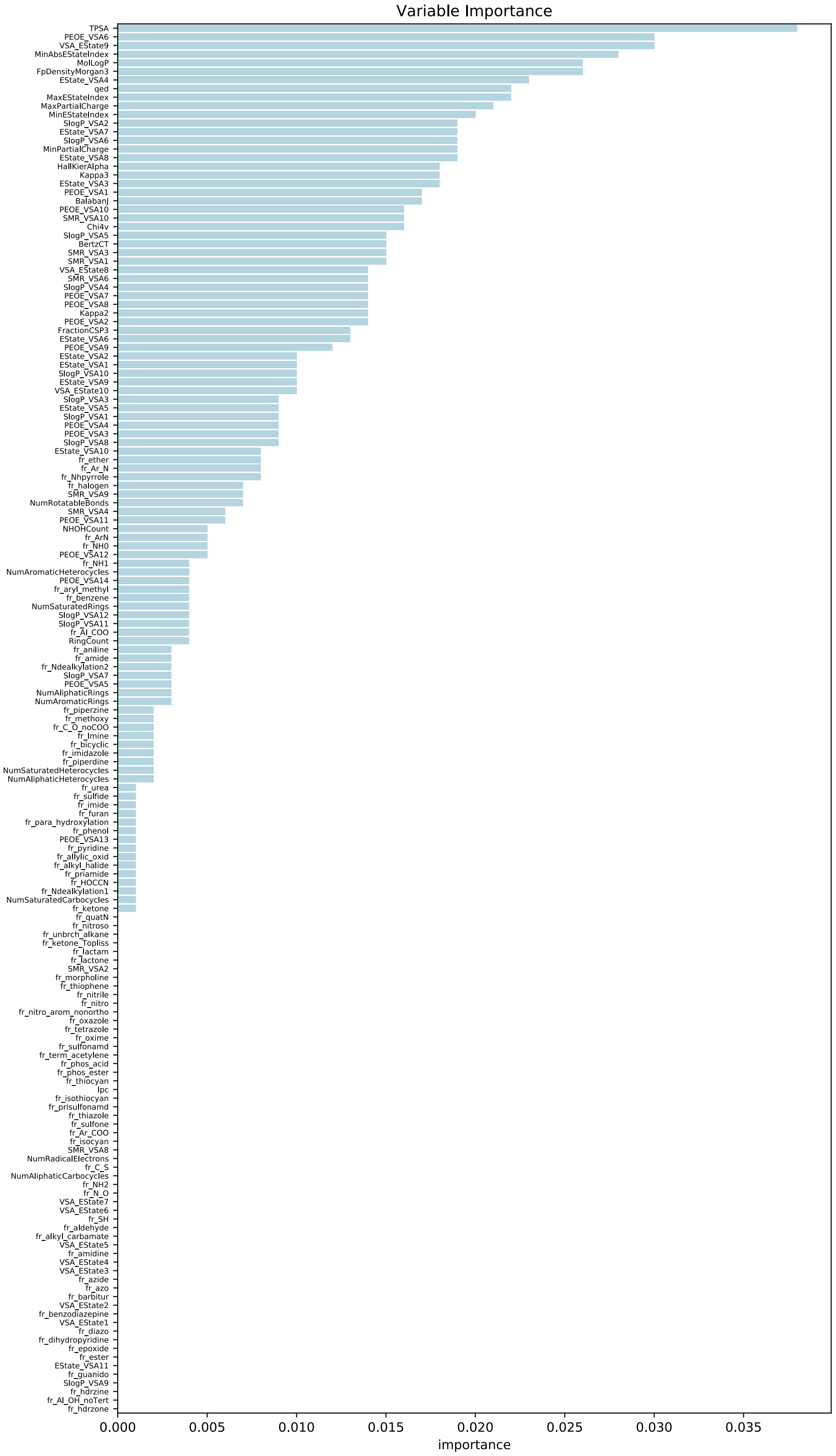

Figure S10. Variables importance for optimized gradient boosting classifier.

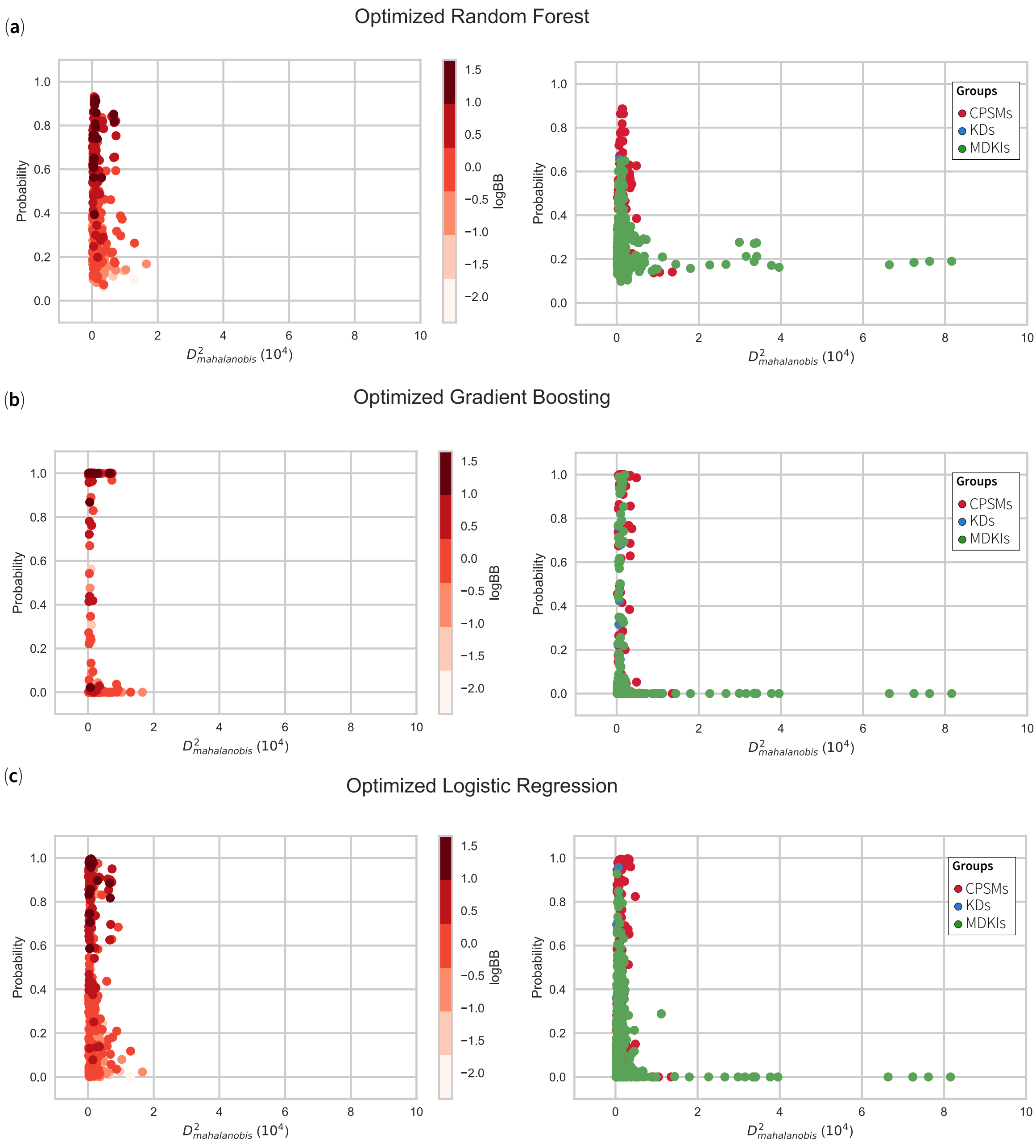

Figure S11. Distribution of model set (left) and holdout set (right) with class 1 probability estimates (BBB+, y-axis) and Mahalanobis squared distance (x-axis) for our 3 models: optimized random forest (**a**), optimized gradient boosting (**b**) and optimized logistic regression (**c**) classifiers. On the model set, logBB values were added in a gradient of reds while the holdout set was divided by groups (CPSMs, KDs, MDKIs).

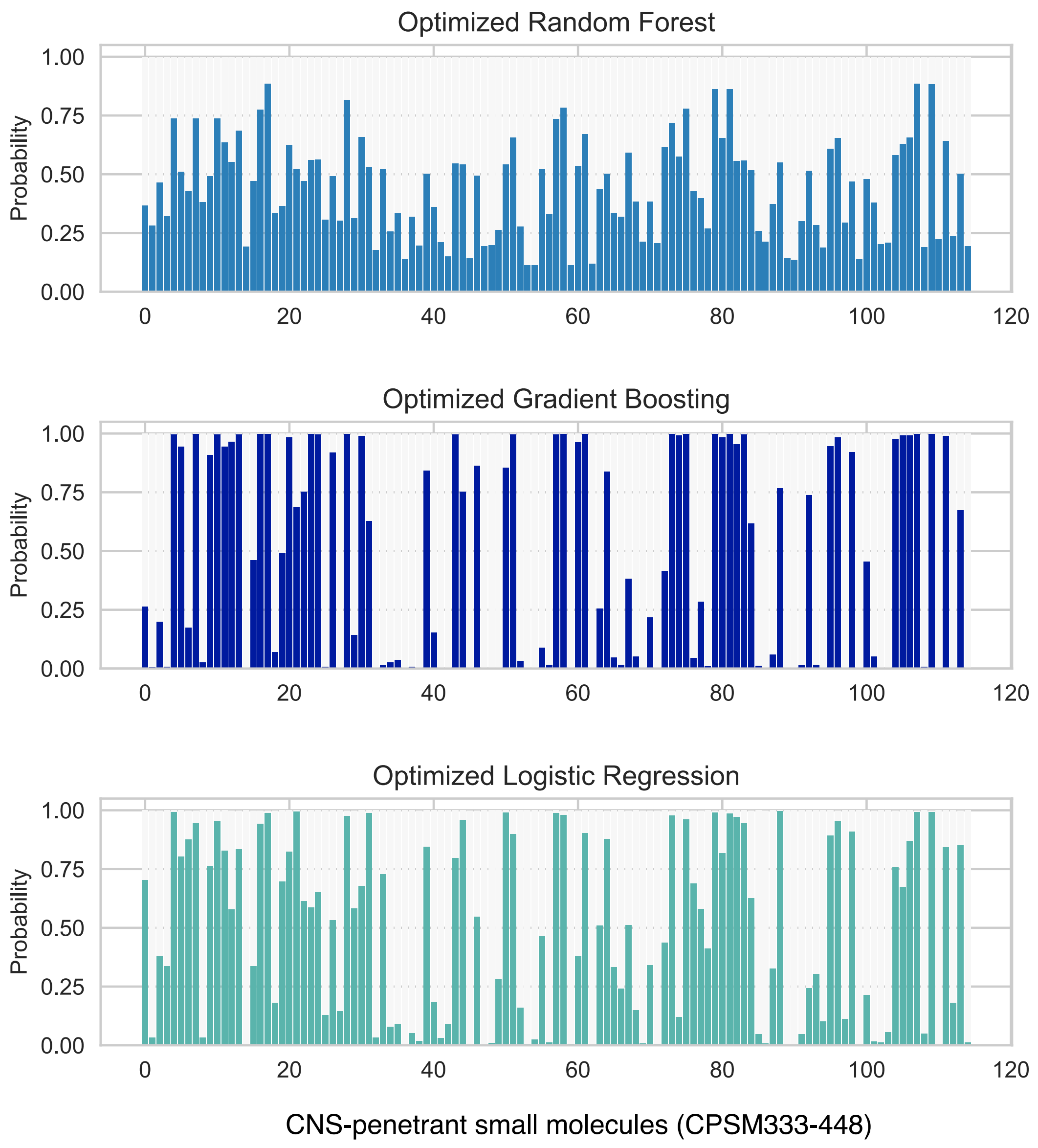

Figure S12. Performance results from optimized binary classifiers (random forest, gradient boosting and logistic regression) for 116 CNS-penetrant small molecules (CPSMs). Y-axis represents the probability to belong to class 1 (logBB>0.1). A compound with a probability value >0.50 is predicted to pass the blood-brain barrier.

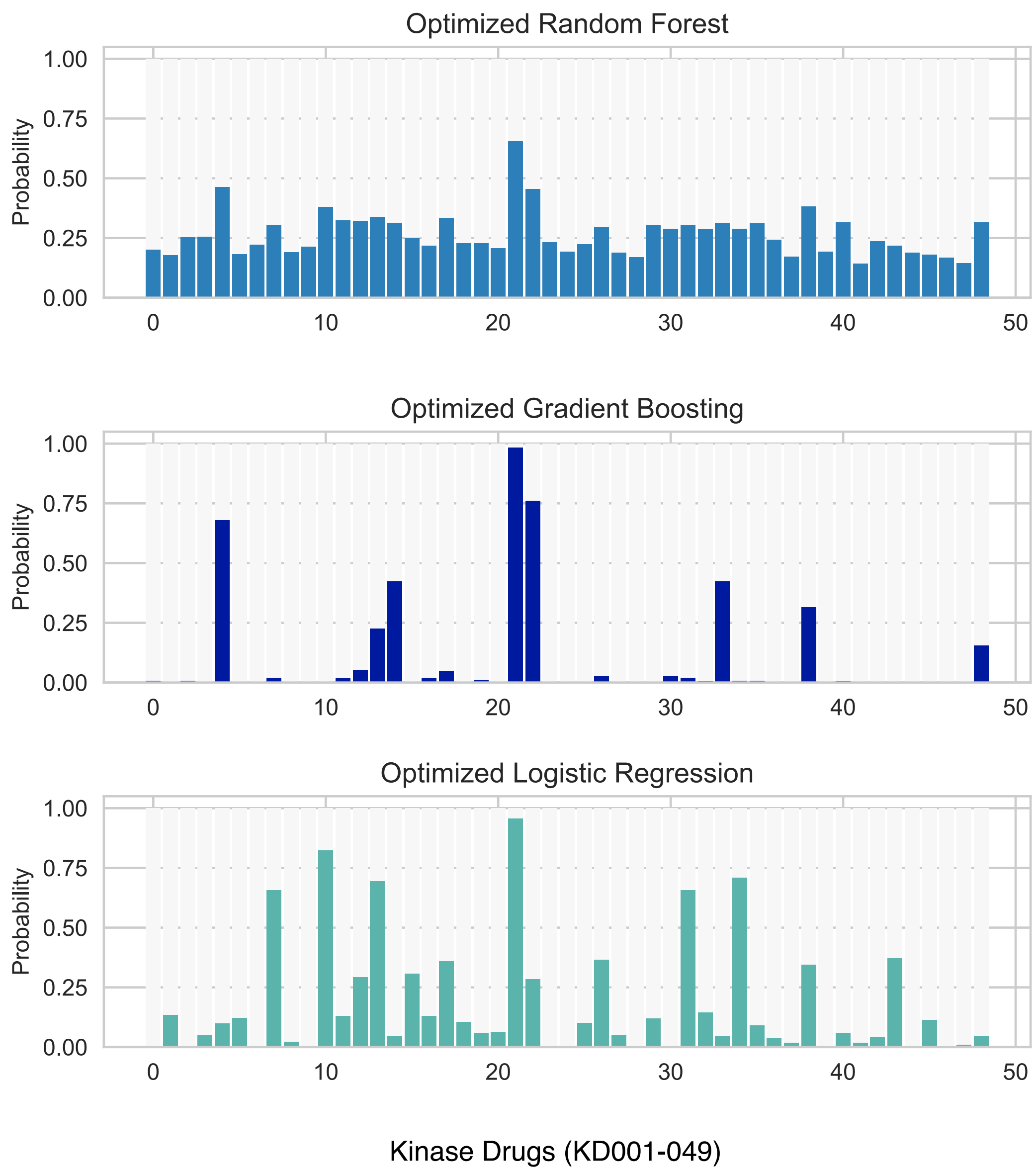

Figure S13. Performance results from optimized binary classifiers (random forest, gradient boosting and logistic regression) for 49 kinase drugs (KDs). Y-axis represents the probability to belong to class 1 (logBB>0.1). A compound with a probability value >0.50 is predicted to pass the blood-brain barrier.

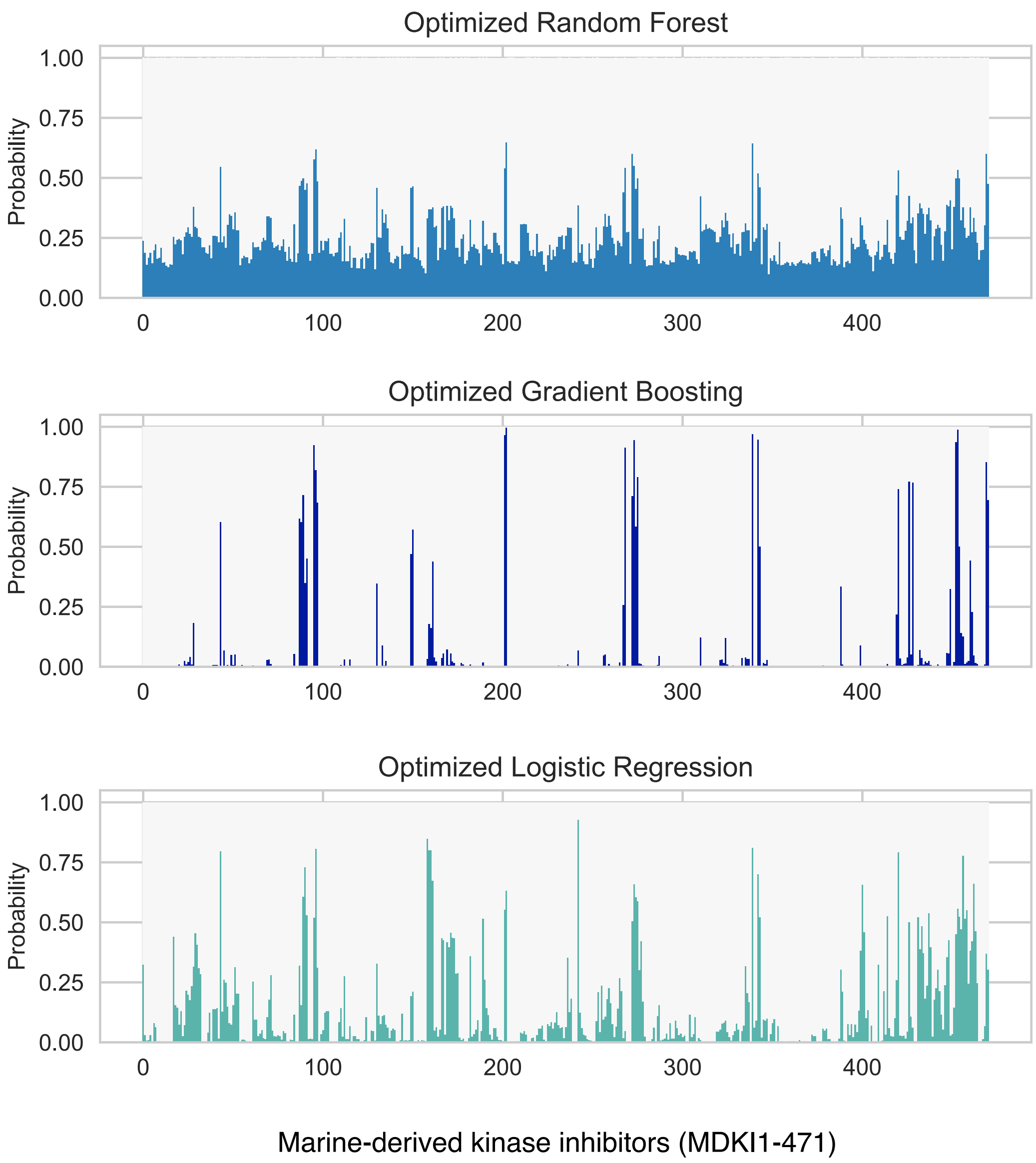

Figure S14. Performance results from optimized binary classifiers (random forest, gradient boosting and logistic regression) for 471 marine-derived kinase inhibitors (MDKIs). Y-axis represents the probability to belong to class 1 (logBB>0.1). A compound with a probability value >0.50 is predicted to pass the blood-brain barrier.
